# DNA Damage and Repair Deficiency in ALS/FTD-Associated Neurodegeneration: From Molecular Mechanisms to Therapeutic Implication

**DOI:** 10.3389/fnmol.2021.784361

**Published:** 2021-12-16

**Authors:** Haibo Wang, Manohar Kodavati, Gavin W. Britz, Muralidhar L. Hegde

**Affiliations:** ^1^Department of Neurosurgery, Center for Neuroregeneration, Houston Methodist Research Institute, Houston, TX, United States; ^2^Department of Neuroscience Research at Neurological Surgery, Weill Medical College, New York, NY, United States

**Keywords:** amyotrophic lateral sclerosis, frontotemporal dementia, FUS, DNA damage, DNA repair deficiency

## Abstract

Emerging studies reveal that neurodegenerative disorders, including amyotrophic lateral sclerosis (ALS) and frontotemporal dementia (FTD), are commonly linked to DNA damage accumulation and repair deficiency. Neurons are particularly vulnerable to DNA damage due to their high metabolic activity, relying primarily on oxidative phosphorylation, which leads to increased reactive oxygen species (ROS) generation and subsequent DNA damage. Efficient and timely repair of such damage is critical for guarding the integrity of genomic DNA and for cell survival. Several genes predominantly associated with RNA/DNA metabolism have been implicated in both ALS and FTD, suggesting that the two diseases share a common underlying pathology with varied clinical manifestations. Recent studies reveal that many of the gene products, including RNA/DNA binding proteins (RBPs) TDP-43 and FUS are involved in diverse DNA repair pathways. A key question in the etiology of the ALS/FTD spectrum of neurodegeneration is the mechanisms and pathways involved in genome instability caused by dysfunctions/mutations of those RBP genes and their consequences in the central nervous system. The understanding of such converging molecular mechanisms provides insights into the underlying etiology of the rapidly progressing neurodegeneration in ALS/FTD, while also revealing novel DNA repair target avenues for therapeutic development. In this review, we summarize the common mechanisms of neurodegeneration in ALS and FTD, with a particular emphasis on the DNA repair defects induced by ALS/FTD causative genes. We also highlight the consequences of DNA repair defects in ALS/FTD and the therapeutic potential of DNA damage repair-targeted amelioration of neurodegeneration.

## Introduction

Neurodegeneration is characterized by an age-associated or pathological progression-induced loss of neuronal structures and functions, eventually causing neuronal death. The four most common neurodegenerative disorders are Alzheimer’s disease (AD), Parkinson’s disease (PD), Huntington’s disease (HD), and amyotrophic lateral sclerosis (ALS). In the motor neuron disease ALS, the degeneration can occur in both upper and lower motor neurons in the brain, brainstem, and spinal cord, which leads to muscle weakness and atrophy (Ragagnin et al., 2019). ALS commonly starts with the early symptoms of weakness in the limb or bulbar muscles and progresses to difficulties in swallowing, speech, and respiration and eventual death due to respiratory failure. ALS is typically late-onset and fatal, with a median incidence of approximately 1–3 cases per hundred thousand persons per year worldwide and a 5–10% survival rate for over 10 years after diagnosis (Vijayakumar et al., 2019; Rojas et al., 2020). Approximately 90% of ALS cases are identified as sporadic (SALS), while ~10% are familial (FALS). In addition to motor dysfunction, up to 50% of ALS patients may also develop cognitive impairments and behavioral deficiencies, and among them, 15–20% are diagnosed with another neurodegenerative disorder, frontotemporal dementia (FTD), one of the most common types of dementia in people under 65 (Zago et al., 2011; Bartoletti et al., 2019; Abramzon et al., 2020). Similarly, up to 30% of FTD patients can develop motor dysfunction (Abramzon et al., 2020).

The etiology of ALS and FTD remains complex. Given the overlap of their clinical and pathological characteristics, the genetic basis of the two disorders has been extensively investigated, and some genes that are highly associated with both diseases have been identified as common genetic causes of ALS and FTD, including chromosome 9 open reading frame 72 (*C9orf72*), fused in sarcoma (*FUS*), and TAR DNA binding protein 43 (*TDP-43*). Among the causative genes, hexanucleotide repeat expansion within a non-coding region of *C9orf72* is one of the most common genetic cause of ALS and FTD (DeJesus-Hernandez et al., 2011; Ling et al., 2013). In this review, we discuss the emerging molecular mechanisms of various subtypes of ALS/FTD, with a particular focus on diverse causative genes and the role of genome damage and repair defects, as well as novel therapeutic avenues.

## Common Mechanisms of Neurodegeneration in ALS and FTD

While both ALS and FTD share several neuropathological, clinical, and genetic attributes, they are distinguished by the region they affect in the central nervous system (CNS), symptoms, and onset age of the disease. Both diseases have been linked to a diverse set of genes that cumulatively cause loss of critical cellular functions and gain of toxicity. For example, RNA binding proteins (RBPs) FUS and TDP-43, are predominantly localized to the nucleus in healthy neurons, with a small fraction shuttling between the nucleus and cytoplasm. However, in ALS/FTD, these RBPs show cytoplasmic aggregate formation due to their nucleo-cytosolic mislocalization. The nuclear clearance of FUS and TDP-43 leads to their loss of function in repairing genome damage in addition to their role in RNA processing. Together with the gain of toxicity in the cytosol likely *via* altered stress granule dynamics, this contributes to neuronal death (Lenzi et al., 2015; Wang H. et al., 2018; Mitra et al., 2019; Wang and Hegde, 2019; Zhang et al., 2020; Ding et al., 2021). A third key genetic factor in ALS/FTD is *C9orf72*, which involves an expanded hexanucleotide repeat (GGGGCC) in its non-coding region (DeJesus-Hernandez et al., 2011). In healthy individuals, the hexanucleotide repeats range from 2 to 24, and expansion above 30 is considered pathogenic. While in ALS/FTD patients, the repeats can range anywhere between 30 and 100 in a small number of cases, and 100–1,000 repeats or even higher in the majority of the cases (DeJesus-Hernandez et al., 2011; Dols-Icardo et al., 2014; Smeyers et al., 2021). Apart from variations in the repeat number among individuals, there is variation in repeat size in the same individual when compared between CNS and blood(Van Mossevelde et al., 2017). Although the actual function of the *C9orf72* encoded protein is not known, the pathogenic repeat expansion in *C9orf72* causes haploinsufficiency as well as a gain of function in the form of aggregating expanded RNAs and dipeptide repeat proteins (Balendra and Isaacs, 2018; Fumagalli et al., 2021).

### Disruption of RNA Transactions/Homeostasis

Dysfunctional RNA metabolism has been recognized as a central pathway in the progression of ALS and FTD, and the identification of RBP dysregulation as a major player in ALS-FTD supports this mechanism. RBP gene mutations including *TDP-43, FUS*, Ataxin-2* (ATXN2)*, RBP EWS (*EWSR1)*, TATA-box binding protein-associated factor 15 *(TAF15)*, heterogeneous nuclear ribonucleoprotein A1 *(hnRNPA1)*, heterogeneous nuclear ribonucleoprotein A2/B1 *(hnRNPA2/B1)*, TIA1 cytotoxic granule associated RNA binding protein* (TIA*1), and matrin3 (*MATR3)* are reported in FALS and FTD cases, which comprises ~10% and 50% of the total cases, respectively (Kabashi et al., 2008; Elden et al., 2010; Ticozzi et al., 2011; Couthouis et al., 2012; Kim et al., 2013; Johnson et al., 2014; Mackenzie et al., 2017). Mutant RBPs may have a diverse effect on RNA homeostasis, including splicing, transport, and editing.

The role of FUS and TDP-43 in RNA splicing is well documented. High-throughput and computational approaches identified ~5,500 RNA targets for FUS in human and mouse brains. FUS affects expression levels of more than 600 genes, and splicing patterns of more than 350 genes are changed, whereas TDP-43 affects the expression of over 100 of the same genes (Lagier-Tourenne et al., 2012). The common feature among these genes is the presence of exceptionally long introns with multiple binding sites for both FUS and TDP-43 (Lagier-Tourenne et al., 2012). FUS interacts in the spliceosome complex with key splicing factors, including Y box binding protein 1 (YB-1), hnRNP A1, and U1 small nuclear ribonucleoprotein (U1 snRNP; Butti and Patten, 2019). FUS is also a part of the minor spliceosome complex and functions in the removal of minor introns. The FUS P525L mutation, causing nuclear clearance, leads to an inhibition in splicing minor introns by mis-localizing minor spliceosome components (Reber et al., 2016).

Furthermore, both FUS and TDP-43 are components of cytoplasmic ribonucleoprotein (RNP) transport granules. In mouse neuronal cultures, FUS is translocated to dendrites as an RNA-protein complex in response to the activation of metabotropic glutamate receptor 5 (mGluR5; Fujii et al., 2005). FUS binds to the mRNA of the actin-stabilizing protein nuclear distribution protein nudE homolog 1 (Ndl-1), and its transcripts are increased in dendrites following mGluR activation, whereas levels of Ndl-1 are significantly reduced in FUS-null dendrites (Sasagawa et al., 2002; Fujii and Takumi, 2005). In neuronal axons, TDP-43 is co-localized with known transport-associated RBPs, including IGF2 mRNA-binding protein-1 (IMP1), fragile X mental retardation protein (FMRP), and ELAV-like protein 4 (HuD; Fallini et al., 2012; Ferro et al., 2018). Cytoplasmic TDP-43 mRNP granules show bidirectional microtubule-dependent transport in neurons, whereas ALS-causing TDP-43 mutations (M337V, G298S, and A315T) impair this function (Alami et al., 2014). Neurofilament light chain (NEFL) mRNA is transported by TDP-43 mRNPs and is defective in mutant motor neurons (Alami et al., 2014). TDP-43 represses splicing of non-conserved cryptic exons; when TDP-43 is depleted, it leads to splicing of cryptic exons into mRNA and translation disruption causing nonsense-mediated decay of mRNA. In TDP-43 ALS-FTD cases, cryptic exon repression was impaired (Ling et al., 2015). In the case of the Neurofascin gene, TDP-43 binding to its mRNA is important for its expression. In TDP-43 loss of function phenotype, a previously unidentified cryptic exon is retained in the neurofascin gene targeting the mRNA to undergo nonsense-mediated decay (Chang et al., 2021). TDP-43 regulates the expression of stathmin-2, a neuronal growth associated factor. Depletion of TDP-43 reduces its binding to the first intron of stathmin-2 pre-mRNA, which uncovers a cryptic polyadenylation site whose retention produces truncated, nonfunctional mRNA. The depletion of TDP-43 by antisense oligonucleotides causes inhibition in axonal regeneration in induced pluripotent cell (iPSC)-derived motor neuron cells, these cells can be rescued by stathmin-2 expression causing restoration of axonal regenerative capacity (Melamed et al., 2019). UNC13A an ALS/FTD risk gene encodes a neuronal protein that functions in the vesicle priming step before synaptic vesicle fusion, mice lacking the protein exhibit functional deficits in glutamatergic synapses (Augustin et al., 1999; Lipstein et al., 2017). Neuronal loss of TDP-43 causes a reduction in UNC13A expression by the inclusion of cryptic exon in UNC13A mRNA. The cryptic exon was also shown to carry a variant associated with ALS/FTD risk in humans (Ma et al., 2021).

### Stress Granules and Protein Inclusions

Stress granules are an important part of RNA metabolism during cellular stress and are composed primarily of RNAs and RBPs. Stress granules can be induced by various cellular stressors, including glucose starvation, oxidative stress, viral infection, and mitochondrial dysfunction (Piotrowska et al., 2010; Fu et al., 2016; Palangi et al., 2017; Moon and Parker, 2018). Once stress is relieved, the stress granule can be either disassembled or degraded by autophagy. Stress granules represent an RNA silencing strategy to conserve energy by shifting translation to produce only essential proteins for survival (Ivanov et al., 2019). Several ALS/FTD associated proteins, including FUS, TDP-43, TIA-1, Ataxin-2, and hnRNPs, are associated with stress granules. As most of these proteins possess long intrinsically disordered domains, failure to resolve the stress granules can lead to protein aggregate formation (Wolozin and Ivanov, 2019; Baradaran-Heravi et al., 2020; Marcelo et al., 2021). Consistently, defective stress granule assembly and disassembly leading to the formation of pathogenic protein aggregates is also linked to neurodegeneration (Protter and Parker, 2016). Ubiquitin-positive protein aggregates are commonly seen in the neuronal cytoplasm of postmortem ALS/FTD cases (Vanden Broeck et al., 2014). The majority of these aggregated proteins belong to the RBP family of proteins, and mutated RBPs account for the majority of familial cases. TDP-43 pathological inclusions are seen in 97% of ALS and 45% of FTD cases, whereas FUS-positive inclusions are seen in 2% of ALS and 9% of FTD cases (Aulas and Vande Velde, 2015; Prasad et al., 2019).

### Autophagy

Due to their postmitotic nature, neurons cannot dilute toxic cell components by cell division, instead, they depend on active protein quality control mechanisms for cell viability and homeostasis. Studies have demonstrated that defects in the induction and clearance of autophagosomes are associated with the pathogenesis of neurodegenerative diseases (Tran and Reddy, 2020). Many ALS-associated genes whose toxicity is related to protein misfolding and aggregation have a connection to the autophagy cascade and function in different stages of autophagy. ALS-associated SOD1 mutations cause defective vesicle nucleation by destabilizing the BECN1-BCL2L1 complex, thereby impacting autophagy stimulation (Nassif et al., 2014). Mutations in p62 (SQSTM1) are reported in the case of FALS and SALS, and Optineurin (OPTN) mutations are seen in rare ALS cases, TANK binding kinase-1 (TBK1) mutations connected to ALS are identified in eight independent genetic screens. Both p62 and OPTN function as autophagy receptors and are important for the initiation step of autophagy (Maruyama et al., 2010; Lattante et al., 2015; Shen et al., 2015; Cui et al., 2018). TBK1 is important for autophagy as it phosphorylates a number of autophagy adaptors, including p62, OPTN, and NDP52, and it is also important for autophagosome maturation (Pilli et al., 2012; Heo et al., 2015). In the maturation stage, an autophagosome fuses with a lysosome. Valosin-containing protein (VCP), an AAA-ATPase whose mutation is associated with ALS, is important for recycling or degradation of ubiquitinated proteins either by the ubiquitin proteasomal or autophagy-mediated lysosomal system. VCP loss leads to the accumulation of immature autophagosomes with ubiquitin-positive substrates (Ju et al., 2009; Johnson et al., 2010; Meyer et al., 2012). FUS and TDP-43 clearance are associated with autophagy, and the pharmacological activation of autophagy leads to increased turnover and early clearance of these proteins from stress granules (Wang et al., 2012; Ryu et al., 2014; Cheng et al., 2015). In the case of C9orf72, wild-type protein interacts with Rab1a preferentially in the GTP-bound state and controls trafficking of ULK1, a kinase involved in autophagosome formation. C9orf72 depletion causes dysregulation of autophagosome function (Farg et al., 2014; Monahan et al., 2016).

### Mitochondrial Dysfunction

Although it is debatable whether it is a cause or an effect, mitochondrial dysfunction is a constant feature in neurodegeneration. Mitochondrial fragmentation, alteration in mitochondrial morphology, fission, and fusion-associated gene expression variations are seen in ALS, along with altered Ca^2+^ metabolism and reactive oxygen species (ROS) generation (Lin and Beal, 2006). Many proteins associated with ALS, including FUS, TDP-43, and SOD1, also localize in mitochondria and affect their dynamics, including mitochondrial fission, fusion, and localization. Mutations or cellular stress-induced increases in TDP-43 expression lead to mitochondrial unfolded protein response (UPRmt) and downregulation of LonP1, a protease involved in the degradation of mitochondrial TDP-43, which leads to severe mitochondrial damage and causes advancement of disease onset in a TDP-43 expressing fly model (Wang P. et al., 2019). FUS interacts with mitochondrial chaperon HSP60. HSP60 expression increase is seen in two out of three FTD-FUS patients, and knockdown of HSP60 causes a decrease in FUS localization to the mitochondria. In a transgenic FUS fly model, RNAi-mediated downregulation of the HSP60 homolog partially rescued the neurodegenerative FUS phenotype (Deng et al., 2015). A FUS mutation associated with ALS/FTD, FUS R521C, causes mitochondrial dysfunction by preferentially sequestering respiratory chain complex mRNAs leading to reduced expression of those proteins. In comparison to wild-type FUS, mutant FUS expressed in mitochondria binds to the mitochondrial mRNAs ND1, CYTB, COX1, and ATP6 five-fold more strongly (Tsai et al., 2020). FUS interacts with mitochondrial ATP synthase 5 beta and disrupts ATP synthase complex assembly (Deng et al., 2018). ALS-associated FUS mutations and FUS ΔNLS cause mitochondrial shortening and fragmentation due to the mutant FUS binding to mature mRNAs and altering the expression of those genes, including mitochondria-associated genes (Nakaya and Maragkakis, 2018). Both FUS and TDP-43 affect endoplasmic reticulum (ER)-mitochondria interaction dynamics, leading to defective Ca^2+^ metabolism, and both proteins do so by activating GSK-3β, which disrupts VAPB-PTPIP51 interaction. The alteration in Ca^2+^ levels causes defects in ATP production (Stoica et al., 2014, 2016). Mitochondrial DNA repair is another frontier, which needs further investigation due to emerging findings that show DNA repair roles of ALS-associated proteins, such as FUS and TDP-43 (Kodavati et al., 2020).

### Nucleocytoplasmic Transport Defects

The nuclear pore complex (NPC), which is central to nucleocytoplasmic transport, disassemble and reassemble during mitosis. In post-mitotic cells, although some components like Nup153 and Nup50 of the complex are continuously exchanged, the scaffold nucleoporins like Nup107/160 remain in the complex. Aging is associated with deterioration of the NPC integrity (D’Angelo et al., 2009). TDP-43 cytoplasmic inclusions are identified in approximately 97% and 45% of ALS and FTD cases, aggregate specific interacting partners of TDP-43 include components of NPC and nucleocytoplasmic transport machinery (Ling et al., 2013; Chou et al., 2018). In Human neurons expressing mutant FUS, reduction in nucleo-cytoplasmic transport and decreased density of nucleoporins (Nups) is observed. FUS and Nups are found to be interacting independent of RNA, FUS-Nup interaction is seen in the nucleus of healthy neurons, whereas in mutant FUS carrying cells, they are seen in the cytoplasm (Lin et al., 2021). In the case of C9orf72 repeat expansion, the toxic dipeptides expressed are shown to be directly binding to the central channel of the nuclear pores and inhibit the transport of macromolecules through the nuclear pore. The binding of these toxic dipeptides to NPC is mediated by polymeric forms of nuclear pore protein and phenylalanine: glycine repeats of dipeptide repeats (Shi et al., 2017).

## DNA Repair Defects and Resulting Genome Instability in ALS/FTD

Genome damage and its defective repair have been etiologically linked to degenerating neurons in various neurodegenerative diseases, including ALS/FTD, however, the specific mechanisms remain enigmatic (Wang et al., 2017). Early studies of ataxia-telangiectasia, a childhood disease affecting the development of the neuron system caused by mutations in ATM, a serine/threonine kinase that plays a critical role in repairing DNA double-strand breaks (DSBs), provided the first linkage between DNA damage and neurodegeneration(Savitsky et al., 1995; Rothblum-Oviatt et al., 2016). Subsequent investigations revealed that several ALS/FTD-linked genes including *FUS, TDP-43, SOD1, C9orf72, Never-in-mitosis A (NIMA) related kinase-1 (NEK1), C21orf2, Senetaxin (SETX), and Valosin containing protein 1 (VCP)* are involved in the DNA damage response (DDR; Acs et al., 2011; Fang et al., 2015; Guerrero et al., 2016; van Rheenen et al., 2016; Higelin et al., 2018; Konopka and Atkin, 2018; Wang H. et al., 2018; Bordoni et al., 2019; Mitra et al., 2019). The mutation, mislocalization, or defects in those genes mediated DNA repair deficiency have been etiologically linked to ALS and FTD (Sun et al., 2020).

### Genomic DNA Damages and Their Repair in the Context of Normal Physiology

Human genomic DNA is continuously damaged by multiple endogenous or exogenous factors, including DNA replication errors, activities of enzymes like DNA glycosylase and topoisomerase, ROS, ultraviolet (UV) radiation, ionizing radiation (IR), and chemicals (Hegde et al., 2008; Chatterjee and Walker, 2017). Depending on the sources, various types of DNA damage are induced, which include several dozen oxidized DNA bases, base mismatches, DNA interstrand crosslinks, and strand breaks, including single-strand breaks (SSBs) and DSBs. The DSBs are the most lethal form of damage, and a single unrepaired DSB may be sufficient to trigger cell death (Chatterjee and Walker, 2017; Trenner and Sartori, 2019). The endogenous factor, ROS, is continuously generated during the cellular metabolisms like ATP production and inflammation response, and thus ROS-induced damage is the most critical threat to the genome of normal tissues, especially in the CNS due to the high consumption of oxygen by the brain (around 20% of the body; Lopez-Gonzalez et al., 2016; Wang et al., 2017). The major forms of ROS include superoxide radical anion (O_2_−), hydroxyl radical (•OH), as well as hydrogen peroxide (H_2_O_2_; Kowalska et al., 2020). The most commonly observed oxidative DNA damage is the 8-oxo-7, 8-dihydroguanine (8-oxoG), which is a •OH mediated C-8 hydroxylation of guanine and is recognized as oxidative DNA damage marker. Notably, 8-oxoG lesion is higher in mitochondrial DNA than in nuclear DNA, suggesting a susceptibility of mitochondrial DNA to oxidative damage (Guillaumet-Adkins et al., 2017). ROS can induce SSBs directly or indirectly (during oxidized lesion repair), and unrepaired adjacent oxidative damage can result in secondary DSBs. It’s worth mentioning that oxidation-related genome damages occur transiently at early-response gene promoters. This neural activity-induced gene expression is essential during the development of the brain and neuron maturation (Madabhushi et al., 2014, 2015; Su et al., 2015). Although the evolutionary significance of such DNA break formations during normal physiological processes is still debated, DNA damage is highly increased in the aging human brain and has been linked to defects in learning, memory, and neuronal survival (Lu et al., 2004).

**Figure 1:**
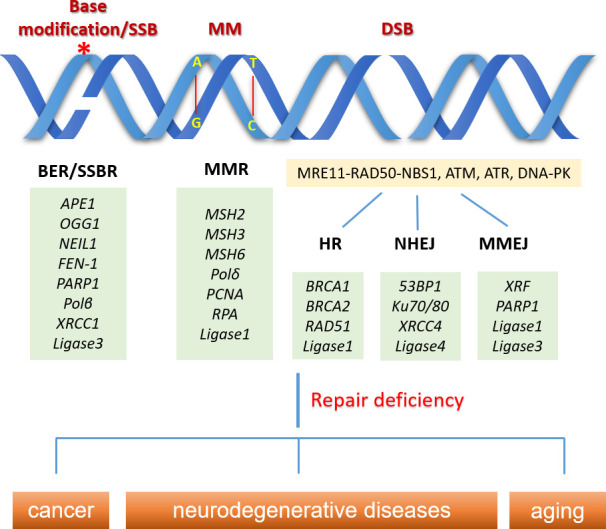
Schematic overview of common DNA damage types and their repair pathways in normal physiology. DNA base modification (e.g., 8-oxo-7, 8-dihydroguanine) can be repaired by base excision repair (BER) initiated with the removal of the oxidized base by specific DNA glycosylases, leading to the generation of single-strand break (SSB), which are eventually resolved by SSB repair (SSBR) pathway involving DNA polymerase β and DNA ligase 3. DNA mismatches (MM) are repaired through the mismatch repair (MMR) pathway. MM can be recognized by a group of very conserved MutS homologs (e.g., MSH2, MSH3, and MSH6), and the DNA altered region is eventually replaced through the action of DNA polymerase δ and DNA ligase 1. Double strand breaks (DSBs) are sensed by MRE11-RAD50-NBS1, ATM, ATR, DNA-PK, while cells can choose different pathways (homologous recombination (HR), nonhomologous end joining (NHEJ), or microhomology-mediated end joining (MMEJ)) to repair the breaks, based on the cell types, cell phases and the structure of the DNA break ends. DNA repair deficiency is linked to human diseases like cancer and neurodegeneration, as well as aging. *Indicates the base modification of DNA.

DNA damage can be both mutagenic and cytotoxic, and the DNA damages that are not repaired efficiently and timely are associated with multiple human diseases, including cancer and neurodegenerations. Oxidative DNA damages are mainly repaired by base excision repair (BER). BER is initiated with the removal of the oxidized base by specific DNA glycosylases, leading to the generation of apurinic/apyrimidinic (AP) site that is further cleaved to form an SSB. The DNA breaks are eventually resolved by DNA polymerase β and DNA ligase 3, which share the same steps of the SSB repair (SSBR) pathway. BER repairs non-bulky DNA lesion, while ROS and UV-induced bulky lesion is repaired by nucleotide excision repair (NER). Although BER and NER share some common repair steps like DNA damage recognition, damaged nucleotide excision, and DNA resynthesis, BER repairs fragments having a length of 1–6 bases, whereas NER is capable of repairing DNA fragments with a length of 30 bases (Lee and Kang, 2019; Casal-Mourino et al., 2020). DNA mismatches are usually generated during DNA replication, which is repaired through the mismatch repair (MMR) pathway. DNA mismatches can be recognized by a group of very conserved MutS homologs (e.g., MSH2, MSH3, and MSH6), and the DNA altered region is eventually replaced through the action of DNA polymerase δ and DNA ligase 1 (Jiricny, 2013). DNA DSBs can be repaired by homologous recombination (HR), nonhomologous end joining (NHEJ), or microhomology-mediated end joining (MMEJ). HR is the most accurate pathway for repairing DSB because the repair is processed based on the sister chromatid and thus, it is only available in the S and G2 phases of dividing cells when the DNA is replicated, but not in the neurons, which is one of the major differences in the DNA repair pathways between dividing and non-dividing cells, although some proteins of the HR pathway are still present in non-dividing cells (Iyama and Wilson, 2013; Fugger and West, 2016). Compared with HR, NHEJ provides an error-prone repair by direct ligating the DSB ends, which exists in all cell cycles and is a major pathway in repairing cellular DSBs. Besides, NHEJ was shown to be critical in maintaining the proliferation of non-dividing cells and is the only active DSB repair pathway in postmitotic cells (Iyama and Wilson, 2013; Baleriola et al., 2016). The alignment of microhomologous sequences between the broken ends differentiates MMEJ from NHEJ (Sfeir and Symington, 2015). MMEJ was initially believed to play a backup role of NHEJ and was less studied, while a recent work showed a more important role of MMEJ as an advantageous choice for cells to quickly repair DSBs to avoid cell death (Truong et al., 2013; Vaidya et al., 2014; Dutta et al., 2017). However, the function of MMEJ in DSB repair in neurons is unclear. DNA interstrand crosslinks are highly toxic due to the covalent connection of the two DNA strands, thereby blocking DNA replication and transcription (Truong et al., 2013). The repair of DNA interstrand crosslink is complicated and involves multiple DNA repair pathways. The interstrand crosslinks occurring outside of S phase are mainly repaired by NER while those occurring in S phase are primarily repaired by Fanconi anemia (FA) pathway, a repair pathway relying on an E3 ubiquitin ligase complex formed by eight FA genes and coordination with HR (McHugh and Sarkar, 2006; Kim and D’Andrea, 2012; Hashimoto et al., 2016; Liu et al., 2020). However, HR was shown to be unessential for DNA interstrand crosslinks repair in quiescent cells (G0/G1 phase), likely because NER plays a dominant role in those cells (Figure 1; Hashimoto et al., 2016).

Multiple biochemical methods are used to investigate DNA damages. Foci formation assay is a very commonly used tool to visualize DNA damage in cells. The assay is based on the posttranslational modification (e.g., phosphorylation), or physical recruitment and accumulation of certain DDR factors (e.g., H2XA, ATM, and 53BP1) at DNA damage termini in response to a DSB, which makes it possible to stain the proteins with fluorescent antibodies and show bright dots as “foci” under a fluorescence microscope. The foci formation assay provides a straightforward way to observe the DSB, and the disappearance of the foci is closely related to the kinetics of DNA damage repair. However, despite the utilization of DSB detection only, DSB independent foci formation of the DDR proteins is a major challenge to the assay (Wang et al., 2014). Single-cell gel electrophoresis assay (also known as comet assay) is a method to measure DNA strand breaks in eukaryotic cells. Depending on the electrophoresis condition, comet assay is subdivided into neutral and alkaline comet assays. The neutral comet assay is mostly used to detect DSBs, whereas alkaline comet assay reveals a phenotype with multiple types of DNA damages, including both DSBs and SSBs (Collins, 2004; Lu et al., 2017). By comparing neutral and alkaline comet assays, DNA damages other than DSBs can be exhibited. However, comet assay does not directly show the number of specific DNA lesions (Moller et al., 2020). Besides, the severity of the DNA damage is reflected by the measuring of the tail length and tail moment of the comet image by software, the outcome may vary when measured with different software (Lu et al., 2017). Long amplicon-polymerase chain reaction (LA-PCR) is another widely used way to assess DNA damages. LA-PCR reveals DNA damage by amplifying a long fragment (around 10kb) of a target gene (like housekeeping genes). The PCR product may be reduced due to the damage of the DNA template compared to undamaged control. Parallel amplification of a shorter fragment within the same target gene is needed to normalize the DNA copy number or to compare DNA damage from different DNA sources (Jung et al., 2009). Although LA-PCR doesn’t differentiate DNA damage types, since it’s a PCR-based measurement, LA-PCR can be directly used to detect DNA damages of cultured cells, fresh or frozen tissues from animal models or patients. LA-PCR can also be used to detect the DNA damage in mitochondria by using mitochondrial DNA-specific primers. Finally, several DNA damage repair reporter systems have also been well developed and established in living cells to study specific DNA damage repair pathways, including MMR, HR, NHEJ, and MMEJ, although it relies on the detectable expression of the reporter constructed plasmids in cultured cells (Wang et al., 2009, 2013; Zhou et al., 2009; Dutta et al., 2017; Mitra et al., 2019).

### ALS/FTD-Associated Proteins Play a Role in DNA Damage Repair

While genome damage has been consistently linked to neurodegeneration, including ALS/FTD, the role of disease-linked genetic factors in causing genome instability was not clearly established until recently. For example. Significantly up-regulated DDR markers, phosphorylated H2AX (γ-H2AX) and ATM (p-ATM), cleaved PARP1, and 53BP1 were shown in the lumbar spinal cord from C9orf72-positive ALS patients (Farg et al., 2017).

#### C9orf72

In motor neurons differentiated from ALS/FTD patient-derived iPSCs containing the GGGGCC repeat in* C9orf72*, oxidative stress, and DNA damage were increased compared with healthy controls (Lopez-Gonzalez et al., 2016). The same group subsequently revealed that the expression of the DNA repair protein Ku80, accompanied with the DSB markers phosphorylated ATM and P53, as well as other downstream proapoptotic proteins, are up-regulated in both the *Drosophila* model expressing poly-GR and in C9orf72 iPSC-derived patient neurons (Lopez-Gonzalez et al., 2019). The study also showed that proapoptotic pathways regulated by ATM and P53 could be suppressed by the partial loss of Ku80 in C9orf72 iPSC-derived neurons. Andrade et al. showed that C9orf72 expansion-encoded dipeptide repeat proteins (DPRs) inhibit multiple DNA DSB repair pathways. In addition, single-strand annealing (SSA) repair, a sub-pathway of MMEJ-mediated DSBR, is impaired partially, which is mediated *via* inhibition of nucleolar protein nucleophosmin (NPM1) by DPRs.

Moreover, levels of the SSA component RAD52 are significantly increased in postmortem brain tissues from ALS/FTD samples with C9orf72 pathology as compared to controls (Andrade et al., 2020). A study from Nihei et al. (2020) revealed that heterogeneous ribonucleoprotein (hnRNP) A3 is depleted from the nucleus and partially mislocalizes to cytoplasmic poly-GA inclusions in C9orf72 patients, and the mislocalization caused by poly-GA leads to increased poly-GA production, which partially depletes pATM and consequently enhances DSB accumulation. R-loop is a structure of hybridized nascent RNA and DNA template strands formed during DNA replication, transcription, and DNA repair. The transient formation and disassembly of R-loop structures are tightly regulated. R-loops can be formed by the collision of replication and transcription machinery or due to the absence of RBP’s covering the nascent RNA, making them available to bind ssDNA. R-loop formation can cause replication stalling and replication fork collapse leading to activation of ATR, when DSB is induced after R-loops, it leads to activation of ataxia telangiectasia mutated (ATM). Failure to resolve R-loops in a timely manner may induce the formation of DSBs, which involves severe genome instability (Niehrs and Luke, 2020). Given that R-loops primarily occur at GC-rich transcription sites and are likely in the hexanucleotide repeat expansion (GGGGCC) of *C9orf72*, it is proposed that C9orf72 promotes persistent R-loop dysfunction (Haeusler et al., 2014), which was eventually demonstrated by studies conducted by Walker et al., showing elevated R-loop levels and R-loop-driven DSBs in C9orf72 expansion-expressing cells (Walker et al., 2017).

#### FUS and TDP-43

**Figure 2:**
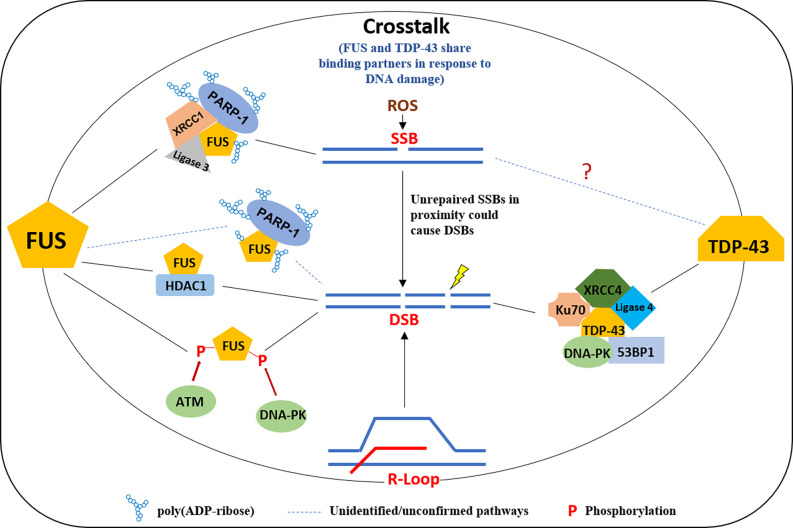
A model of multifaceted involvement of FUS and TDP-43 in the DNA damage response. FUS is recruited to DNA damage sites in a PARP1 activity-dependent manner in response to DNA SSB and forms a complex with XRCC1 and Ligase 3 and is required for ligation activity of Ligase 3. FUS is also involved in DSB repair by interacting HDAC1 or the phosphorylation by ATM/DNA-PK. TDP-43 plays a scaffold protein role at DSB in binding with XRCC4, Ligase 4, Ku70, DNA-PK, and 53BP1, for an efficient DNA repair in NHEJ. FUS, fused in sarcoma; TDP, TAR DNA binding protein; PARP, poly(ADP-ribose) polymerase; DSB, double-strand break.

There is growing evidence that DNA damage is accumulated in FUS and TDP-43 related ALS and FTD patients. γH2AX was found to be significantly increased in brain tissues from patients with neuronal intermediate filament inclusion disease (NIFID), a subtype of FTD-FUS, and in the motor cortex of FALS cases harboring R521C and P525L mutations in FUS (Wang et al., 2013; Higelin et al., 2016). Similarly, increased DNA damage was seen in the frontal cortex sections from patients with FTLD-TDP-43, the spinal cord of ALS patients carrying the TDP43 Q331K mutation, as well as in fibroblasts derived from a TDP-43 ALS patient with M337V mutation (Guerrero et al., 2019; Konopka et al., 2020; Wu et al., 2020). Both FUS and TDP-43 play important roles in DNA damage repair (Figure 2). FUS can be multi-phosphorylated by the DDR kinases ATM and DNA-PK in response to DNA damage (Gardiner et al., 2008; Deng et al., 2014; Rhoads et al., 2018). FALS patients with FUS R521C and P525L mutations show increased DNA damage and the impairment of HR and NHEJ, and a reduced DDR was found in FUS knockdown cells (Wang et al., 2013). An RT^2^ PCR array-based screening reveals distinct perturbations in DDR signaling in FUS-associated motor neuron disease (Wang H. et al., 2019). A number of studies have explored the molecular mechanisms of FUS involving DDR. Coordination between FUS and Poly (ADP-ribose) polymerase (PARP1) is critical for the DNA repair function of FUS. FUS interacts with Poly (ADP-ribose; PAR) and can be PARylated by PARP1, and the inhibition of PARP1 activity prevents the recruitment of FUS onto micro-laser-induced DNA damage tracks (Mastrocola et al., 2013; Rulten et al., 2014). The direct interaction between FUS, PARP1, XRCC1, and Ligase 3 is important for optimal SSB repair (Wang H. et al., 2018). FUS facilitates PARP1-dependent recruitment of the XRCC1-Ligase 3 complex to oxidized genome sites and activates Ligase 3 through direct interaction (Wang H. et al., 2018). Liquid-liquid phase separation is a phenomenon of forming subcellular non-membrane-bound organelles, such as stress granules, as part of the cellular stress response signaling (Wheeler et al., 2016). FUS forms liquid compartments at DNA damage sites and in the cytoplasm upon stress in a PAR interaction-dependent manner, while the ALS-FUS mutant can promote the aggregation of FUS protein-mediated liquid droplets, a common hallmark of FUS pathology in ALS and FTD (Patel et al., 2015). Singatulina et al. (2019) showed that, following PARP-1 activation, FUS binds to PAR and facilitates the formation of damaged DNA-rich compartments to facilitate DNA damage repair, which can be dissociated by Poly(ADP-ribose) glycohydrolase (PARG). Levone et al. (2021) found that liquid-liquid phase separation depends on FUS, which is required for activation of DDR and DSB repair complex assembly. Our and other recent studies demonstrated that TDP-43 also plays an important role in DDR, primarily for the repair of DSBs (Mitra and Hegde, 2019; Mitra et al., 2019; Singatulina et al., 2019). In healthy neurons, TDP-43 is required for the optimal repair of DSBs *via* NHEJ by facilitating the recruitment of the XRCC4-DNA Ligase 4 complex to damage sites. TDP-43 also forms a complex with several DSB repair and response factors, including Ku, DNA-PKcs, 53BP1, and XRCC4-DNA Ligase 4. An ALS-associated TDP-43 mutation prevents nuclear translocation of XRCC4-DNA Ligase 4 complex and is linked to DNA damage mediated neuronal apoptosis (Guerrero et al., 2019). Subsequent studies confirmed the role of TDP-43 in DSB repair pathways in cellular models (Mitra et al., 2019; Konopka et al., 2020). Both FUS and TDP-43 are likely involved in R-loop associated DDR. FUS localizes to the sites of transcription-associated DNA damage and function in the prevention/repair of R-loops (Hill et al., 2016), while TDP-43 mutation was shown to disturb R-loop homeostasis and contributes to R-loop-mediated DNA damage (Giannini et al., 2020). Furthermore, FUS and TDP-43 may crosstalk in DDR. A study showed that TDP-43 and FUS share binding partners after treatment with a DNA damage agent, etoposide, and gene ontology revealed a complex network of interactors with both TDP-43 and FUS. The largest group of interactors shared by FUS and TDP-43 was that of ribosomal proteins, including mitochondria-specific ribosomes (Kawaguchi et al., 2020).

#### SOD1

As the byproduct of normal cellular respiration, ROS is the major source of oxidative stress contributing to DNA damage in neurons. Common ROS include superoxide (O^2−^) and hydrogen peroxide (H_2_O_2_), which are removed/reused by mechanisms involving superoxide dismutases (SOD), catalases, thioredoxin, and glutathione (Tsang et al., 2014). SOD1 is one of three superoxide dismutase family members. SOD1 pathological inclusions are seen in both ALS and FTD cases. SOD1 mutations are only associated with familial ALS but not FTD cases (Vance et al., 2006). In a motor neuron injury mouse model, the human FALS SOD1 G93A mutant accumulates greater DNA damage in the injured motor neurons compared with control, accelerates injury-induced degeneration of the motor neurons, and converts the apoptotic phenotype to a necrotic phenotype, a phenotype showing the pathological process of cell death (Martin et al., 2005). SOD1 is a nuclear transcription factor that regulates oxidative stress resistance. Studies revealed that SOD1 is translocated to the nucleus in an ATM/CDS1-dependent way to regulate gene transcription that is involved in DDR or ROS defense (Tsang et al., 2014; Sun et al., 2020). Dysfunction of SOD1 in ALS not only causes the accumulation of ROS but also affects DDR signaling, a double hit in destabilizing genome integrity.

Crosstalk between SOD1 and other ALS-linked genes has been explored by a number of studies. Abundant inclusions containing misfolded wild-type SOD1 were found in spinal and cortical motor neurons in ALS patients carrying other ALS-causing genes, including the mutations of *C9orf72* and* FUS* (Forsberg et al., 2019). Pokrishevsky et al. observed misfolded SOD1 in perikarya and motor axons of SOD1-FALS and in motor axons of FUS FALS and SALS with TDP-43 pathology, and the overexpression of both wild-type or mutant TDP-43 or the mutant FUS in the cytoplasm can cause misfolding of wild-type SOD1 (Pokrishevsky et al., 2012, 2016). The DNA damage accumulation/DNA repair deficiency in ALS is likely a consequence of interference in DNA damage repair pathways by a combination of factors. Although the detailed mechanisms of this crosstalk require extensive future investigation, Li et al. have demonstrated that cytoplasmic restriction of the mutant SOD1 G93A, which inhibits the nucleic translocation of wild-type SOD1, was directly related to the elevation of DNA damage, partially due to inhibition of the localization of FUS (but not TDP-43) and its related DNA repair enzymes, including HDAC1 and APEX1, in the nuclei (Li et al., 2019).

#### NEK1

NEK1 is a mitotic protein kinase whose heterozygous mutations are associated with ALS. NEK1 localizes to sites of IR-induced DNA damage foci and is a key factor in the early DNA damage repair pathway. NEK1 is important for activation of checkpoint kinases 1 and 2 (CHK1 and CHK2), NEK1 deficiency causes severe alterations in checkpoint control and DNA damage accumulation leading to genome instability (Higelin et al., 2018). NEK1-ALS iPSC motor neurons showed increased γH2AX in comparison to controls, ALS-associated mutations in NEK1 cause haploinsufficiency leading to elevated DNA damage and motor neuron death (Chen et al., 2008; Higelin et al., 2018). NEK1 is also shown to phosphorylate and activate HR repair protein RAD54, which leads to Rad51 removal from chromatin and causes degradation of stalled replication forks (Spies et al., 2016).

#### Other ALS/FTD-Associated Proteins

VCP is a central component of the ubiquitin proteasome system, and mutations in VCP are shown to be associated with ALS. VCP functions in the removal of lysine 48 ubiquitin conjugates from DNA damage sites, making them available for DNA repair factors recruitment (Johnson et al., 2010). In response to DNA damage along with ubiquitin ligase RNF8, VCP facilitates recruitment of 53BP1, BRCA1, and RAD51 factors critical for DNA repair and survival (Meerang et al., 2011). In C9ORF72 ALS, depletion of VCP impairs 53BP1 recruitment to DNA damage sites (Walker and El-Khamisy, 2018). *C21orf2* is identified as an ALS-associated gene by genome-wide association study and meta-analysis, it is known to form a functional complex with NEK1, and both proteins stabilize each other (van Rheenen et al., 2016; Watanabe et al., 2020). C21orf2 deficient cells show less efficient DNA repair than control cells, NEK1 overexpression can complement C21orf2 depletion. Only the HR pathway requires C21orf2/NEK1 complex, NHEJ repair can function without this complex (Fang et al., 2015). Mutations in the SETX gene are responsible for a dominantly inherited form of ALS, which has early onset (Groh et al., 2017). SETX gene encodes an RNA/DNA helicase involved in DNA damaged response, SETX is recruited to DNA DSBs at transcriptionally active sites. Upon binding, SETX promotes Rad51 recruitment and prevents mutagenic NHEJ (Cohen et al., 2018; Rawal et al., 2020). Exome sequencing analysis identified mutations in Matrin3, an RNA binding protein associated with familial ALS (Boehringer et al., 2017). ALS associated Matrin3 mutation causes its altered re-distribution in the nucleus and alters mRNA nuclear export, Matrin3 knockdown causes a reduction in RAD51 protein levels leading to HR disturbances and increased radiation sensitivity (Boehringer et al., 2017). Matrin3 interacts with PSF, a splicing factor that interacts with RAD51 and modulates its activity in HR depletion of Matrin3 causes prolonged retention of PSF at DNA damage sites (Salton et al., 2010; Boehringer et al., 2017). ALS-associated mutations are identified in low complexity domains of hnRNPA1, it plays an important role in maintaining the integrity of telomeres (Sui et al., 2015). Phosphorylation of hnRNPA1 promotes a switch from replication protein A (RPA) to the protection of telomeres 1 (POT1) at newly replicated telomeric overhangs (Sui et al., 2015).

### Potential Consequences of DNA Repair Defects in ALS/FTD

**Figure 3:**
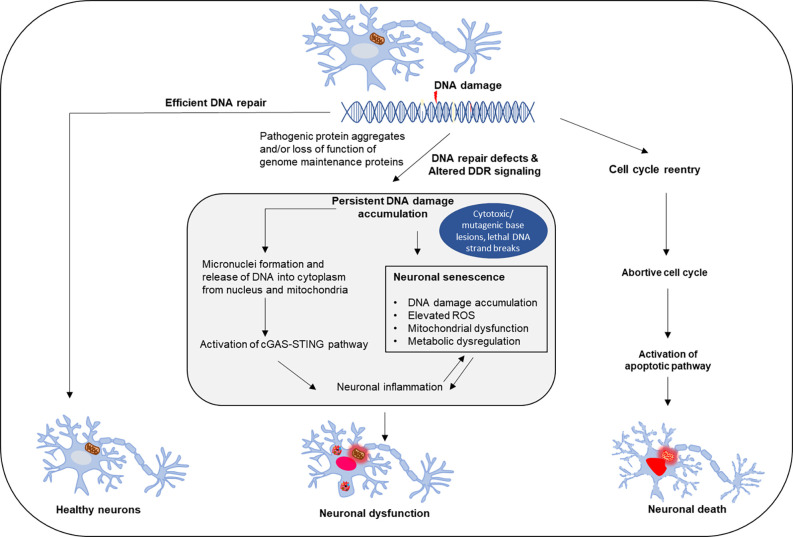
Effect of DNA repair defects on neuronal fate. Neuronal genomes are continuously challenged by damage that occurs frequently due to endogenous or exogenous causes. Healthy neurons require efficient and timely DNA repair, while DNA damage accumulation and repair deficiency induce neuronal dysfunction and degeneration. The consequences of neuronal DNA repair defects and deregulation in DNA damage response include inflammation and neuronal senescence, as well as pathological cell cycle reentry of postmitotic neurons, which triggers apoptosis-mediated neuronal cell death.

DDR and DNA repair deficiency are widely recognized as common premises in many neurodegenerative disorders. Mutations in DNA repair and DDR genes associated with neurodegenerative diseases including mutations in NER genes associated with Cockayne syndrome, Xeroderma Pigmentosum, and Trichothiodystrophy, mutations in BER genes associated with ataxia oculomotor apraxia, and DSB repair gene mutations causing ataxia telangiectasia, XLF syndrome, and Ligase 4 syndrome (Le Ber et al., 2003; Kraemer et al., 2007; Chistiakov, 2010; Keimling et al., 2011). Although the link between DNA damage and neurodegeneration is generally accepted, mechanisms of neuronal DNA repair and implications of such repair defects are still not fully understood, which is a roadblock for developing effective DNA repair-based therapeutics. Notably, there could be subtle differences in the preferences for various DNA repair pathways and vulnerability to the DNA damage sources at different stages of neuronal development and maturity. One consequence of the robust increase in the unrepaired genome in postmitotic neurons is accidental cell cycle re-entry, presumably to activate cell cycle-associated DNA repair pathways, but with a catastrophic outcome, as this can cause neuronal cell death by apoptosis (Folch et al., 2012; Figure 3). Furthermore, ALS-affected neurons show many classical features of cellular senescence, including senescence-associated neuroinflammation, increased DNA damage, activated DDR, metabolic dysregulation, mitochondrial dysfunction, and elevated ROS levels (Martinez-Cue and Rueda, 2020). Recent studies have identified the cGAS-STING pathway as a link between DNA damage and inflammation (Decout et al., 2021). The formation of micronuclei is associated with DNA damage, and the nuclear envelope of micronuclei is ruptured to reveal DNA in the cytoplasm, which causes activation of cGAS (Kwon et al., 2020). In TDP-43 mutant mice and iPSC-derived motor neurons, recruitment of TDP-43 to mitochondria causes the release of DNA through the permeability transition pore leading to activation of the cGAS-STING pathway (Yu et al., 2020). Elevated levels of cGMP, a secondary messenger in the cGAS-STING pathway, are seen in patients’ spinal cords and activation of this pathway can be a critical determinant of TDP-43 pathology (Yu et al., 2020). Thus, DNA damage defects could directly contribute to neuronal degeneration or disturb other mechanisms that maintain normal cellular functions (Figure 3).

## Future Direction: Targeting DNA Damage for ALS-FTD Treatment

Although the available treatments can transiently slow down the progression of the ALS-FTD or can help manage some symptoms, there is currently no cure for these diseases. Identification of the involvement of several ALS-FTD causative genes in DDR raises the possibility of targeting DNA damage as a therapeutic strategy. Growing evidence has shown that neurodegeneration can be prevented by rescuing DNA damage repair deficiency, as supported by studies in cultured cells or animal models. Lopez-Gonzalez et al. (2019) found that the expression of protein Ku80, required for NHEJ-mediated DSB repair, was greatly elevated in *Drosophila* expressing poly-GR and in C9orf72 iPSC-derived FTD patient neurons, and partial inhibition of Ku80 suppressed the cell death of the neurons. In another study, Walker et al. (2017) revealed that defective ATM activation induced by the expression of C9orf72 expansions is a consequence of the accumulation of p62, and the depletion of p62 can restore the defective ATM-mediated DNA repair which suggests a therapeutic opportunity for C9orf72-related neuropathologies. Decreased ligation activity of Ligase 3 and Ligase 4 were seen in FUS mutant, and TDP-43 depleted cells, respectively (Wang H. et al., 2018; Mitra et al., 2019). Identifying ways to target and rescue the DNA ligation activity of these ligases in FUS- and TDP-43-associated ALS-FTD patients could lead to new treatment avenues for these diseases. In response to DNA damage, PARP1 is activated by its auto-PARylation, which then PARylates a number of target proteins to activate or recruit DDR factors to repair the damage. In a study conducted by McGurk et al. (2018), significantly elevated levels of nuclear PAR were seen in the postmortem spinal cord from ALS patients with phosphorylated TDP-43 inclusions, indicating that the PARPs are highly activated. They further found that in the cytoplasm, the activity of PARP 1/2 regulates stress granule formation and stress-induced TDP-43 aggregation, which can be suppressed by the PARP 1/2 inhibitor veliparib. Finally, they found that veliparib mitigates TDP-43 toxicity in primary spinal cord neurons isolated from rat embryos. Neurodegeneration in ALS/FTD could be a consequence of increased DNA damage, DNA damage repair deficiency, or a cumulative effect of both. A therapeutic strategy targeting DNA damage for ALS/FTD treatment likely can be reached by either improving DNA damage repair efficiency or alleviating the toxic events that induce DNA damage in neurons.

## Author Contributions

HW and MK prepared the manuscript with inputs from MH, who provided supervision. GB provided critical comments and discussions. All authors contributed to the article and approved the submitted version.

## Conflict of Interest

The authors declare that the research was conducted in the absence of any commercial or financial relationships that could be construed as a potential conflict of interest.

## Publisher’s Note

All claims expressed in this article are solely those of the authors and do not necessarily represent those of their affiliated organizations, or those of the publisher, the editors and the reviewers. Any product that may be evaluated in this article, or claim that may be made by its manufacturer, is not guaranteed or endorsed by the publisher.

## References

[B1] Abramzon Y. A. Fratta P. Traynor B. J. Chia R. (2020). The overlapping genetics of amyotrophic lateral sclerosis and frontotemporal dementia. Front. Neurosci. 14:42. 10.3389/fnins.2020.00042 32116499 PMC7012787

[B2] Acs K. Luijsterburg M. S. Ackermann L. Salomons F. A. Hoppe T. Dantuma N. P. (2011). The AAA-ATPase VCP/p97 promotes 53BP1 recruitment by removing L3MBTL1 from DNA double-strand breaks. Nat. Struct. Mol. Biol. 18, 1345–1350. 10.1038/nsmb.2188 22120668

[B3] Alami N. H. Smith R. B. Carrasco M. A. Williams L. A. Winborn C. S. Han S. S. W.. (2014). Axonal transport of TDP-43 mRNA granules is impaired by ALS-causing mutations. Neuron 81, 536–543. 10.1016/j.neuron.2013.12.018 24507191 PMC3939050

[B4] Andrade N. S. Ramic M. Esanov R. Liu W. Rybin M. J. Gaidosh G.. (2020). Dipeptide repeat proteins inhibit homology-directed DNA double strand break repair in C9ORF72 ALS/FTD. Mol. Neurodegener. 15:13. 10.1186/s13024-020-00365-9 32093728 PMC7041170

[B5] Augustin I. Rosenmund C. Sudhof T. C. Brose N. (1999). Munc13-1 is essential for fusion competence of glutamatergic synaptic vesicles. Nature 400, 457–461. 10.1038/22768 10440375

[B6] Aulas A. Vande Velde C. (2015). Alterations in stress granule dynamics driven by TDP-43 and FUS: a link to pathological inclusions in ALS? Front. Cell. Neurosci. 9:423. 10.3389/fncel.2015.00423 26557057 PMC4615823

[B7] Balendra R. Isaacs A. M. (2018). C9orf72-mediated ALS and FTD: multiple pathways to disease. Nat. Rev. Neurol. 14, 544–558. 10.1038/s41582-018-0047-2 30120348 PMC6417666

[B8] Baleriola J. Alvarez-Lindo N. de la Villa P. Bernad A. Blanco L. Suarez T.. (2016). Increased neuronal death and disturbed axonal growth in the Polmu-deficient mouse embryonic retina. Sci. Rep. 6:25928. 10.1038/srep25928 27172884 PMC4865816

[B9] Baradaran-Heravi Y. Van Broeckhoven C. van der Zee J. (2020). Stress granule mediated protein aggregation and underlying gene defects in the FTD-ALS spectrum. Neurobiol. Dis. 134:104639. 10.1016/j.nbd.2019.104639 31626953

[B10] Bartoletti M. Bosco D. A. Da Cruz S. Lagier-Tourenne C. Liachko N. Markmiller S.. (2019). Phenotypic suppression of ALS/FTD-associated neurodegeneration highlights mechanisms of dysfunction. J. Neurosci. 39, 8217–8224. 10.1523/JNEUROSCI.1159-19.2019 31619490 PMC6794934

[B11] Boehringer A. Garcia-Mansfield K. Singh G. Bakkar N. Pirrotte P. Bowser R. (2017). ALS associated mutations in matrin 3 alter protein-protein interactions and impede mRNA nuclear export. Sci. Rep. 7:14529. 10.1038/s41598-017-14924-6 29109432 PMC5674072

[B12] Bordoni M. Pansarasa O. Dell’Orco M. Crippa V. Gagliardi S. Sproviero D.. (2019). Nuclear phospho-SOD1 protects DNA from oxidative stress damage in amyotrophic lateral sclerosis. J. Clin. Med. 8:729. 10.3390/jcm8050729 31121901 PMC6572067

[B13] Butti Z. Patten S. A. (2019). RNA dysregulation in amyotrophic lateral sclerosis. Front. Genet. 9:712. 10.3389/fgene.2018.00712 30723494 PMC6349704

[B14] Casal-Mourino A. Ruano-Ravina A. Torres-Duran M. Parente-Lamelas I. Provencio-Pulla M. Castro-Anon O.. (2020). Polymorphisms in the BER and NER pathways and their influence on survival and toxicity in never-smokers with lung cancer. Sci. Rep. 10:21147. 10.1038/s41598-020-78051-5 33273562 PMC7713126

[B15] Chang K. J. Agrawal I. Vainshtein A. Ho W. Y. Xin W. Tucker-Kellogg G.. (2021). TDP-43 maximizes nerve conduction velocity by repressing a cryptic exon for paranodal junction assembly in Schwann cells. eLife 10:e64456. 10.7554/eLife.64456 33689679 PMC7946431

[B16] Chatterjee N. Walker G. C. (2017). Mechanisms of DNA damage, repair and mutagenesis. Environ. Mol. Mutagen. 58, 235–263. 10.1002/em.22087 28485537 PMC5474181

[B17] Chen Y. Chen P. L. Chen C. F. Jiang X. Riley D. J. (2008). Never-in-mitosis related kinase 1 functions in DNA damage response and checkpoint control. Cell Cycle 7, 3194–3201. 10.4161/cc.7.20.6815 18843199 PMC2597191

[B18] Cheng C. W. Lin M. J. Shen C. K. (2015). Rapamycin alleviates pathogenesis of a new Drosophila model of ALS-TDP. J. Neurogenet. 29, 59–68. 10.3109/01677063.2015.1077832 26219309

[B19] Chistiakov D. A. (2010). Ligase IV syndrome. Adv. Exp. Med. Biol. 685, 175–185. 10.1007/978-1-4419-6448-9_16 20687505

[B20] Chou C. C. Zhang Y. Umoh M. E. Vaughan S. W. Lorenzini I. Liu F.. (2018). TDP-43 pathology disrupts nuclear pore complexes and nucleocytoplasmic transport in ALS/FTD. Nat. Neurosci. 21, 228–239. 10.1038/s41593-017-0047-3 29311743 PMC5800968

[B21] Cohen S. Puget N. Lin Y. L. Clouaire T. Aguirrebengoa M. Rocher V.. (2018). Senataxin resolves RNA:DNA hybrids forming at DNA double-strand breaks to prevent translocations. Nat. Commun. 9:533. 10.1038/s41467-018-02894-w 29416069 PMC5803260

[B22] Collins A. R. (2004). The comet assay for DNA damage and repair: principles, applications and limitations. Mol. Biotechnol. 26, 249–261. 10.1385/MB:26:3:249 15004294

[B23] Couthouis J. Hart M. P. Erion R. King O. D. Diaz Z. Nakaya T.. (2012). Evaluating the role of the FUS/TLS-related gene EWSR1 in amyotrophic lateral sclerosis. Hum. Mol. Genet. 21, 2899–2911. 10.1093/hmg/dds116 22454397 PMC3373238

[B24] Cui R. Tuo M. Li P. Zhou C. (2018). Association between TBK1 mutations and risk of amyotrophic lateral sclerosis/frontotemporal dementia spectrum: a meta-analysis. Neurol. Sci. 39, 811–820. 10.1007/s10072-018-3246-0 29349657

[B25] D’Angelo M. A. Raices M. Panowski S. H. Hetzer M. W. (2009). Age-dependent deterioration of nuclear pore complexes causes a loss of nuclear integrity in postmitotic cells. Cell 136, 284–295. 10.1016/j.cell.2008.11.037 19167330 PMC2805151

[B26] Decout A. Katz J. D. Venkatraman S. Ablasser A. (2021). The cGAS-STING pathway as a therapeutic target in inflammatory diseases. Nat. Rev. Immunol. 21, 548–569. 10.1038/s41577-021-00524-z 33833439 PMC8029610

[B27] DeJesus-Hernandez M. Mackenzie I. R. Boeve B. F. Boxer A. L. Baker M. Rutherford N. J.. (2011). Expanded GGGGCC hexanucleotide repeat in noncoding region of C9ORF72 causes chromosome 9p-linked FTD and ALS. Neuron 72, 245–256. 10.1016/j.neuron.2011.09.011 21944778 PMC3202986

[B30] Deng Q. Holler C. J. Taylor G. Hudson K. F. Watkins W. Gearing M.. (2014). FUS is phosphorylated by DNA-PK and accumulates in the cytoplasm after DNA damage. J. Neurosci. 34, 7802–7813. 10.1523/JNEUROSCI.0172-14.2014 24899704 PMC4044245

[B28] Deng J. Wang P. Chen X. Cheng H. Liu J. Fushimi K.. (2018). FUS interacts with ATP synthase beta subunit and induces mitochondrial unfolded protein response in cellular and animal models. Proc. Natl. Acad. Sci. U S A 115, E9678–E9686. 10.1073/pnas.1806655115 30249657 PMC6187197

[B29] Deng J. Yang M. Chen Y. Chen X. Liu J. Sun S.. (2015). FUS interacts with HSP60 to promote mitochondrial damage. PLoS Genet. 11:e1005357. 10.1371/journal.pgen.1005357 26335776 PMC4559378

[B31] Ding Q. Chaplin J. Morris M. J. Hilliard M. A. Wolvetang E. Ng D. C. H.. (2021). TDP-43 mutation affects stress granule dynamics in differentiated NSC-34 motoneuron-like cells. Front. Cell Dev. Biol. 9:611601. 10.3389/fcell.2021.611601 34169068 PMC8217991

[B32] Dols-Icardo O. Garcia-Redondo A. Rojas-Garcia R. Sanchez-Valle R. Noguera A. Gomez-Tortosa E.. (2014). Characterization of the repeat expansion size in C9orf72 in amyotrophic lateral sclerosis and frontotemporal dementia. Hum. Mol. Genet. 23, 749–754. 10.1093/hmg/ddt460 24057670

[B33] Dutta A. Eckelmann B. Adhikari S. Ahmed K. M. Sengupta S. Pandey A.. (2017). Microhomology-mediated end joining is activated in irradiated human cells due to phosphorylation-dependent formation of the XRCC1 repair complex. Nucleic Acids Res. 45, 2585–2599. 10.1093/nar/gkw1262 27994036 PMC5389627

[B34] Elden A. C. Kim H. J. Hart M. P. Chen-Plotkin A. S. Johnson B. S. Fang X.. (2010). Ataxin-2 intermediate-length polyglutamine expansions are associated with increased risk for ALS. Nature 466, 1069–1075. 10.1038/nature09320 20740007 PMC2965417

[B35] Fallini C. Bassell G. J. Rossoll W. (2012). The ALS disease protein TDP-43 is actively transported in motor neuron axons and regulates axon outgrowth. Hum. Mol. Genet. 21, 3703–3718. 10.1093/hmg/dds205 22641816 PMC3406762

[B36] Fang X. Lin H. Wang X. Zuo Q. Qin J. Zhang P. (2015). The NEK1 interactor, C21ORF2, is required for efficient DNA damage repair. Acta Biochim. Biophys. Sin. (Shanghai) 47, 834–841. 10.1093/abbs/gmv076 26290490 PMC4581587

[B37] Farg M. A. Sundaramoorthy V. Sultana J. M. Yang S. Atkinson R. A. Levina V.. (2014). C9ORF72, implicated in amytrophic lateral sclerosis and frontotemporal dementia, regulates endosomal trafficking. Hum. Mol. Genet. 23, 3579–3595. 10.1093/hmg/ddu068 24549040 PMC4049310

[B38] Farg M. A. Konopka A. Soo K. Y. Ito D. Atkin J. D. (2017). The DNA damage response (DDR) is induced by the C9orf72 repeat expansion in amyotrophic lateral sclerosis. Hum. Mol. Genet. 26, 2882–2896. 10.1093/hmg/ddx170 28481984

[B39] Ferro D. Yao S. Zarnescu D. C. (2018). Dynamic duo - FMRP and TDP-43: Regulating common targets, causing different diseases. Brain Res. 1693, 37–42. 10.1016/j.brainres.2018.04.034 29715444 PMC5997554

[B40] Folch J. Junyent F. Verdaguer E. Auladell C. Pizarro J. G. Beas-Zarate C.. (2012). Role of cell cycle re-entry in neurons: a common apoptotic mechanism of neuronal cell death. Neurotox. Res. 22, 195–207. 10.1007/s12640-011-9277-4 21965004

[B41] Forsberg K. Graffmo K. Pakkenberg B. Weber M. Nielsen M. Marklund S.. (2019). Misfolded SOD1 inclusions in patients with mutations in C9orf72 and other ALS/FTD-associated genes. J. Neurol. Neurosurg. Psychiatry 90, 861–869. 10.1136/jnnp-2018-319386 30992335 PMC6691870

[B42] Fu X. Gao X. Ge L. Cui X. Su C. Yang W.. (2016). Malonate induces the assembly of cytoplasmic stress granules. FEBS Lett. 590, 22–33. 10.1002/1873-3468.12049 26787461

[B43] Fugger K. West S. C. (2016). Keeping homologous recombination in check. Cell Res. 26, 397–398. 10.1038/cr.2016.25 26902288 PMC4822126

[B44] Fujii R. Okabe S. Urushido T. Inoue K. Yoshimura A. Tachibana T.. (2005). The RNA binding protein TLS is translocated to dendritic spines by mGluR5 activation and regulates spine morphology. Curr. Biol. 15, 587–593. 10.1016/j.cub.2005.01.058 15797031

[B45] Fujii R. Takumi T. (2005). TLS facilitates transport of mRNA encoding an actin-stabilizing protein to dendritic spines. J. Cell Sci. 118, 5755–5765. 10.1242/jcs.02692 16317045

[B46] Fumagalli L. Young F. L. Boeynaems S. De Decker M. Mehta A. R. Swijsen A.. (2021). C9orf72-derived arginine-containing dipeptide repeats associate with axonal transport machinery and impede microtubule-based motility. Sci. Adv. 7:eabg3013. 10.1126/sciadv.abg3013 33837088 PMC8034861

[B47] Gardiner M. Toth R. Vandermoere F. Morrice N. A. Rouse J. (2008). Identification and characterization of FUS/TLS as a new target of ATM. Biochem. J. 415, 297–307. 10.1042/BJ20081135 18620545

[B48] Giannini M. Bayona-Feliu A. Sproviero D. Barroso S. I. Cereda C. Aguilera A. (2020). TDP-43 mutations link Amyotrophic Lateral Sclerosis with R-loop homeostasis and R loop-mediated DNA damage. PLoS Genet. 16:e1009260. 10.1371/journal.pgen.1009260 33301444 PMC7755276

[B49] Groh M. Albulescu L. O. Cristini A. Gromak N. (2017). Senataxin: genome guardian at the interface of transcription and neurodegeneration. J. Mol. Biol. 429, 3181–3195. 10.1016/j.jmb.2016.10.021 27771483

[B51] Guerrero E. N. Mitra J. Wang H. Rangaswamy S. Hegde P. M. Basu P.. (2019). Amyotrophic lateral sclerosis-associated TDP-43 mutation Q331K prevents nuclear translocation of XRCC4-DNA ligase 4 complex and is linked to genome damage-mediated neuronal apoptosis. Hum. Mol. Genet. 28, 2459–2476. 10.1093/hmg/ddz062 31067307 PMC6659010

[B50] Guerrero E. N. Wang H. Mitra J. Hegde P. M. Stowell S. E. Liachko N. F.. (2016). TDP-43/FUS in motor neuron disease: complexity and challenges. Prog. Neurobiol. 145–146, 78–97. 10.1016/j.pneurobio.2016.09.004 27693252 PMC5101148

[B52] Guillaumet-Adkins A. Yanez Y. Peris-Diaz M. D. Calabria I. Palanca-Ballester C. Sandoval J. (2017). Epigenetics and oxidative stress in aging. Oxid. Med. Cell. Longev. 2017:9175806. 10.1155/2017/9175806 28808499 PMC5541801

[B53] Haeusler A. R. Donnelly C. J. Periz G. Simko E. A. Shaw P. G. Kim M. S.. (2014). C9orf72 nucleotide repeat structures initiate molecular cascades of disease. Nature 507, 195–200. 10.1038/nature13124 24598541 PMC4046618

[B54] Hashimoto S. Anai H. Hanada K. (2016). Mechanisms of interstrand DNA crosslink repair and human disorders. Genes Environ. 38:9. 10.1186/s41021-016-0037-9 27350828 PMC4918140

[B55] Hegde M. L. Hazra T. K. Mitra S. (2008). Early steps in the DNA base excision/single-strand interruption repair pathway in mammalian cells. Cell Res. 18, 27–47. 10.1038/cr.2008.8 18166975 PMC2692221

[B56] Heo J. M. Ordureau A. Paulo J. A. Rinehart J. Harper J. W. (2015). The PINK1-PARKIN mitochondrial ubiquitylation pathway drives a program of OPTN/NDP52 recruitment and TBK1 activation to promote mitophagy. Mol. Cell 60, 7–20. 10.1016/j.molcel.2015.08.016 26365381 PMC4592482

[B57] Higelin J. Catanese A. Semelink-Sedlacek L. L. Oeztuerk S. Lutz A. K. Bausinger J.. (2018). NEK1 loss-of-function mutation induces DNA damage accumulation in ALS patient-derived motoneurons. Stem Cell Res. 30, 150–162. 10.1016/j.scr.2018.06.005 29929116

[B58] Higelin J. Demestre M. Putz S. Delling J. P. Jacob C. Lutz A. K.. (2016). FUS mislocalization and vulnerability to DNA damage in ALS patients derived hiPSCs and aging motoneurons. Front. Cell. Neurosci. 10:290. 10.3389/fncel.2016.00290 28082870 PMC5183648

[B59] Hill S. J. Mordes D. A. Cameron L. A. Neuberg D. S. Landini S. Eggan K.. (2016). Two familial ALS proteins function in prevention/repair of transcription-associated DNA damage. Proc. Natl. Acad. Sci. U S A 113, E7701–E7709. 10.1073/pnas.1611673113 27849576 PMC5137757

[B60] Ivanov P. Kedersha N. Anderson P. (2019). Stress granules and processing bodies in translational control. Cold Spring Harb. Perspect. Biol. 11:a032813. 10.1101/cshperspect.a032813 30082464 PMC6496347

[B61] Iyama T. Wilson D. M. 3rd (2013). DNA repair mechanisms in dividing and non-dividing cells. DNA Repair (Amst) 12, 620–636. 10.1016/j.dnarep.2013.04.015 23684800 PMC3720834

[B62] Jiricny J. (2013). Postreplicative mismatch repair. Cold Spring Harb. Perspect. Biol. 5:a012633. 10.1101/cshperspect.a012633 23545421 PMC3683899

[B63] Johnson J. O. Mandrioli J. Benatar M. Abramzon Y. Van Deerlin V. M. Trojanowski J. Q.. (2010). Exome sequencing reveals VCP mutations as a cause of familial ALS. Neuron 68, 857–864. 10.1016/j.neuron.2010.11.036 21145000 PMC3032425

[B64] Johnson J. O. Pioro E. P. Boehringer A. Chia R. Feit H. Renton A. E.. (2014). Mutations in the Matrin 3 gene cause familial amyotrophic lateral sclerosis. Nat. Neurosci. 17, 664–666. 10.1038/nn.3688 24686783 PMC4000579

[B65] Ju J. S. Fuentealba R. A. Miller S. E. Jackson E. Piwnica-Worms D. Baloh R. H.. (2009). Valosin-containing protein (VCP) is required for autophagy and is disrupted in VCP disease. J. Cell Biol. 187, 875–888. 10.1083/jcb.200908115 20008565 PMC2806317

[B66] Jung D. Cho Y. Meyer J. N. Di Giulio R. T. (2009). The long amplicon quantitative PCR for DNA damage assay as a sensitive method of assessing DNA damage in the environmental model, Atlantic killifish (*Fundulus heteroclitus*). Comp. Biochem. Physiol. C Toxicol. Pharmacol. 149, 182–186. 10.1016/j.cbpc.2008.07.007 18706522 PMC2676791

[B67] Kabashi E. Valdmanis P. N. Dion P. Spiegelman D. McConkey B. J. Vande Velde C.. (2008). TARDBP mutations in individuals with sporadic and familial amyotrophic lateral sclerosis. Nat. Genet. 40, 572–574. 10.1038/ng.132 18372902

[B68] Kawaguchi T. Rollins M. G. Moinpour M. Morera A. A. Ebmeier C. C. Old W. M.. (2020). Changes to the TDP-43 and FUS Interactomes Induced by DNA Damage. J. Proteome Res. 19, 360–370. 10.1021/acs.jproteome.9b00575 31693373 PMC6947635

[B69] Keimling M. Volcic M. Csernok A. Wieland B. Dork T. Wiesmuller L. (2011). Functional characterization connects individual patient mutations in ataxia telangiectasia mutated (ATM) with dysfunction of specific DNA double-strand break-repair signaling pathways. FASEB J. 25, 3849–3860. 10.1096/fj.11-185546 21778326

[B71] Kim H. D’Andrea A. D. (2012). Regulation of DNA cross-link repair by the Fanconi anemia/BRCA pathway. Genes Dev. 26, 1393–1408. 10.1101/gad.195248.112 22751496 PMC3403008

[B70] Kim H. J. Kim N. C. Wang Y. D. Scarborough E. A. Moore J. Diaz Z.. (2013). Mutations in prion-like domains in hnRNPA2B1 and hnRNPA1 cause multisystem proteinopathy and ALS. Nature 495, 467–473. 10.1038/nature11922 23455423 PMC3756911

[B72] Kodavati M. Wang H. Hegde M. L. (2020). Altered Mitochondrial dynamics in motor neuron disease: an emerging perspective. Cells 9:1065. 10.3390/cells9041065 32344665 PMC7226538

[B73] Konopka A. Atkin J. D. (2018). The emerging role of DNA damage in the pathogenesis of the C9orf72 repeat expansion in amyotrophic lateral sclerosis. Int. J. Mol. Sci. 19:3137. 10.3390/ijms19103137 30322030 PMC6213462

[B74] Konopka A. Whelan D. R. Jamali M. S. Perri E. Shahheydari H. Toth R. P.. (2020). Impaired NHEJ repair in amyotrophic lateral sclerosis is associated with TDP-43 mutations. Mol. Neurodegener. 15:51. 10.1186/s13024-020-00386-4 32907630 PMC7488163

[B75] Kowalska M. Piekut T. Prendecki M. Sodel A. Kozubski W. Dorszewska J. (2020). Mitochondrial and nuclear DNA oxidative damage in physiological and pathological aging. DNA Cell Biol. 39, 1410–1420. 10.1089/dna.2019.5347 32315547

[B76] Kraemer K. H. Patronas N. J. Schiffmann R. Brooks B. P. Tamura D. DiGiovanna J. J. (2007). Xeroderma pigmentosum, trichothiodystrophy and Cockayne syndrome: a complex genotype-phenotype relationship. Neuroscience 145, 1388–1396. 10.1016/j.neuroscience.2006.12.020 17276014 PMC2288663

[B77] Kwon M. Leibowitz M. L. Lee J. H. (2020). Small but mighty: the causes and consequences of micronucleus rupture. Exp. Mol. Med. 52, 1777–1786. 10.1038/s12276-020-00529-z 33230251 PMC8080619

[B78] Lagier-Tourenne C. Polymenidou M. Hutt K. R. Vu A. Q. Baughn M. Huelga S. C.. (2012). Divergent roles of ALS-linked proteins FUS/TLS and TDP-43 intersect in processing long pre-mRNAs. Nat. Neurosci. 15, 1488–1497. 10.1038/nn.3230 23023293 PMC3586380

[B79] Lattante S. de Calbiac H. Le Ber I. Brice A. Ciura S. Kabashi E. (2015). Sqstm1 knock-down causes a locomotor phenotype ameliorated by rapamycin in a zebrafish model of ALS/FTLD. Hum. Mol. Genet. 24, 1682–1690. 10.1093/hmg/ddu580 25410659

[B80] Le Ber I. Moreira M. C. Rivaud-Pechoux S. Chamayou C. Ochsner F. Kuntzer T.. (2003). Cerebellar ataxia with oculomotor apraxia type 1: clinical and genetic studies. Brain 126, 2761–2772. 10.1093/brain/awg283 14506070

[B81] Lee T. H. Kang T. H. (2019). DNA oxidation and excision repair pathways. Int. J. Mol. Sci. 20:6092. 10.3390/ijms20236092 31816862 PMC6929053

[B82] Lenzi J. De Santis R. de Turris V. Morlando M. Laneve P. Calvo A.. (2015). ALS mutant FUS proteins are recruited into stress granules in induced pluripotent stem cell-derived motoneurons. Dis. Model Mech. 8, 755–766. 10.1242/dmm.020099 26035390 PMC4486861

[B83] Levone B. R. Lenzken S. C. Antonaci M. Maiser A. Rapp A. Conte F.. (2021). FUS-dependent liquid-liquid phase separation is important for DNA repair initiation. J. Cell Biol. 220:e202008030. 10.1083/jcb.202008030 33704371 PMC7953258

[B84] Li J. Song M. Moh S. Kim H. Kim D. H. (2019). Cytoplasmic restriction of mutated SOD1 impairs the DNA repair process in spinal cord neurons. Cells 8:1502. 10.3390/cells8121502 31771229 PMC6952796

[B85] Lin M. T. Beal M. F. (2006). Mitochondrial dysfunction and oxidative stress in neurodegenerative diseases. Nature 443, 787–795. 10.1038/nature05292 17051205

[B86] Lin Y. C. Kumar M. S. Ramesh N. Anderson E. N. Nguyen A. T. Kim B.. (2021). Interactions between ALS-linked FUS and nucleoporins are associated with defects in the nucleocytoplasmic transport pathway. Nat. Neurosci. 24, 1077–1088. 10.1038/s41593-021-00859-9 34059832 PMC8832378

[B87] Ling J. P. Pletnikova O. Troncoso J. C. Wong P. C. (2015). TDP-43 repression of nonconserved cryptic exons is compromised in ALS-FTD. Science 349, 650–655. 10.1126/science.aab0983 26250685 PMC4825810

[B88] Ling S. C. Polymenidou M. Cleveland D. W. (2013). Converging mechanisms in ALS and FTD: disrupted RNA and protein homeostasis. Neuron 79, 416–438. 10.1016/j.neuron.2013.07.033 23931993 PMC4411085

[B89] Lipstein N. Verhoeven-Duif N. M. Michelassi F. E. Calloway N. van Hasselt P. M. Pienkowska K.. (2017). Synaptic UNC13A protein variant causes increased neurotransmission and dyskinetic movement disorder. J. Clin. Invest. 127, 1005–1018. 10.1172/JCI90259 28192369 PMC5330740

[B90] Liu W. Palovcak A. Li F. Zafar A. Yuan F. Zhang Y. (2020). Fanconi anemia pathway as a prospective target for cancer intervention. Cell Biosci. 10:39. 10.1186/s13578-020-00401-7 32190289 PMC7075017

[B91] Lopez-Gonzalez R. Lu Y. Gendron T. F. Karydas A. Tran H. Yang D. (2016). Poly(GR) in C9ORF72-related ALS/FTD compromises mitochondrial function and increases oxidative stress and DNA damage in iPSC-derived motor neurons. Neuron 92, 383–391. 10.1016/j.neuron.2016.09.015 27720481 PMC5111366

[B92] Lopez-Gonzalez R. Yang D. Pribadi M. Kim T. S. Krishnan G. Choi S. Y.. (2019). Partial inhibition of the overactivated Ku80-dependent DNA repair pathway rescues neurodegeneration in C9ORF72-ALS/FTD. Proc. Natl. Acad. Sci. U S A 116, 9628–9633. 10.1073/pnas.1901313116 31019093 PMC6511021

[B93] Lu T. Pan Y. Kao S. Y. Li C. Kohane I. Chan J.. (2004). Gene regulation and DNA damage in the ageing human brain. Nature 429, 883–891. 10.1038/nature02661 15190254

[B94] Lu Y. Liu Y. Yang C. (2017). Evaluating *in vitro* DNA damage using comet assay. J. Vis. Exp. 128:56450. 10.3791/56450 29053680 PMC5752397

[B95] Ma X. R. Prudencio M. Koike Y. Vatsavayai S. C. Kim G. Harbinski F.. (2021). TDP-43 represses cryptic exon inclusion in FTD/ALS gene UNC13A. BioRxiv [Preprint]. 10.1101/2021.04.02.438213 PMC8891019 35197626

[B96] Mackenzie I. R. Nicholson A. M. Sarkar M. Messing J. Purice M. D. Pottier C.. (2017). TIA1 mutations in amyotrophic lateral sclerosis and frontotemporal dementia promote phase separation and alter stress granule dynamics. Neuron 95, 808–816.e9. 10.1016/j.neuron.2017.07.025 28817800 PMC5576574

[B97] Madabhushi R. Gao F. Pfenning A. R. Pan L. Yamakawa S. Seo J.. (2015). Activity-induced DNA breaks govern the expression of neuronal early-response genes. Cell 161, 1592–1605. 10.1016/j.cell.2015.05.032 26052046 PMC4886855

[B98] Madabhushi R. Pan L. Tsai L. H. (2014). DNA damage and its links to neurodegeneration. Neuron 83, 266–282. 10.1016/j.neuron.2014.06.034 25033177 PMC5564444

[B99] Marcelo A. Koppenol R. de Almeida L. P. Matos C. A. Nobrega C. (2021). Stress granules, RNA-binding proteins and polyglutamine diseases: too much aggregation? Cell Death Dis. 12:592. 10.1038/s41419-021-03873-8 34103467 PMC8187637

[B100] Martin L. J. Chen K. Liu Z. (2005). Adult motor neuron apoptosis is mediated by nitric oxide and Fas death receptor linked by DNA damage and p53 activation. J. Neurosci. 25, 6449–6459. 10.1523/JNEUROSCI.0911-05.2005 16000635 PMC6725285

[B101] Martinez-Cue C. Rueda N. (2020). Cellular senescence in neurodegenerative diseases. Front. Cell Neurosci. 14:16. 10.3389/fncel.2020.00016 32116562 PMC7026683

[B102] Maruyama H. Morino H. Ito H. Izumi Y. Kato H. Watanabe Y.. (2010). Mutations of optineurin in amyotrophic lateral sclerosis. Nature 465, 223–226. 10.1038/nature08971 20428114

[B103] Mastrocola A. S. Kim S. H. Trinh A. T. Rodenkirch L. A. Tibbetts R. S. (2013). The RNA-binding protein fused in sarcoma (FUS) functions downstream of poly(ADP-ribose) polymerase (PARP) in response to DNA damage. J. Biol. Chem. 288, 24731–24741. 10.1074/jbc.M113.497974 23833192 PMC3750169

[B104] McGurk L. Mojsilovic-Petrovic J. Van Deerlin V. M. Shorter J. Kalb R.G. Lee V. M.. (2018). Nuclear poly(ADP-ribose) activity is a therapeutic target in amyotrophic lateral sclerosis. Acta Neuropathol. Commun. 6:84. 10.1186/s40478-018-0586-1 30157956 PMC6114235

[B105] McHugh P. J. Sarkar S. (2006). DNA interstrand cross-link repair in the cell cycle: a critical role for polymerase zeta in G1 phase. Cell Cycle 5, 1044–1047. 10.4161/cc.5.10.2763 16687932

[B106] Meerang M. Ritz D. Paliwal S. Garajova Z. Bosshard M. Mailand N.. (2011). The ubiquitin-selective segregase VCP/p97 orchestrates the response to DNA double-strand breaks. Nat. Cell Biol. 13, 1376–1382. 10.1038/ncb2367 22020440

[B107] Melamed Z. Lopez-Erauskin J. Baughn M. W. Zhang O. Drenner K. Sun Y.. (2019). Premature polyadenylation-mediated loss of stathmin-2 is a hallmark of TDP-43-dependent neurodegeneration. Nat. Neurosci. 22, 180–190. 10.1038/s41593-018-0293-z 30643298 PMC6348009

[B108] Meyer H. Bug M. Bremer S. (2012). Emerging functions of the VCP/p97 AAA-ATPase in the ubiquitin system. Nat. Cell Biol. 14, 117–123. 10.1038/ncb2407 22298039

[B109] Mitra J. Guerrero E. N. Hegde P. M. Liachko N. F. Wang H. Vasquez V.. (2019). Motor neuron disease-associated loss of nuclear TDP-43 is linked to DNA double-strand break repair defects. Proc. Natl. Acad. Sci. U S A 116, 4696–4705. 10.1073/pnas.1818415116 30770445 PMC6410842

[B110] Mitra J. Hegde M.L. (2019). A commentary on TDP-43 and DNA damage response in amyotrophic lateral sclerosis. J. Exp. Neurosci. 13:1179069519880166. 10.1177/1179069519880166 31656396 PMC6791036

[B111] Moller P. Azqueta A. Boutet-Robinet E. Koppen G. Bonassi S. Milic M.. (2020). Minimum information for reporting on the comet assay (MIRCA): recommendations for describing comet assay procedures and results. Nat. Protoc. 15, 3817–3826. 10.1038/s41596-020-0398-1 33106678 PMC7688437

[B112] Monahan Z. Shewmaker F. Pandey U. B. (2016). Stress granules at the intersection of autophagy and ALS. Brain Res. 1649, 189–200. 10.1016/j.brainres.2016.05.022 27181519 PMC5055418

[B113] Moon S. L. Parker R. (2018). Analysis of eIF2B bodies and their relationships with stress granules and P-bodies. Sci. Rep. 8:12264. 10.1038/s41598-018-30805-y 30115954 PMC6095920

[B114] Nakaya T. Maragkakis M. (2018). Amyotrophic Lateral Sclerosis associated FUS mutation shortens mitochondria and induces neurotoxicity. Sci. Rep. 8:15575. 10.1038/s41598-018-33964-0 30349096 PMC6197261

[B115] Nassif M. Valenzuela V. Rojas-Rivera D. Vidal R. Matus S. Castillo K.. (2014). Pathogenic role of BECN1/Beclin 1 in the development of amyotrophic lateral sclerosis. Autophagy 10, 1256–1271. 10.4161/auto.28784 24905722 PMC4203551

[B116] Niehrs C. Luke B. (2020). Regulatory R-loops as facilitators of gene expression and genome stability. Nat. Rev. Mol. Cell Biol. 21, 167–178. 10.1038/s41580-019-0206-3 32005969 PMC7116639

[B117] Nihei Y. Mori K. Werner G. Arzberger T. Zhou Q. Khosravi B.. (2020). Poly-glycine-alanine exacerbates C9orf72 repeat expansion-mediated DNA damage *via* sequestration of phosphorylated ATM and loss of nuclear hnRNPA3. Acta Neuropathol. 139, 99–118. 10.1007/s00401-019-02082-0 31642962 PMC6942035

[B118] Palangi F. Samuel S. M. Thompson I. R. Triggle C. R. Emara M. M. (2017). Effects of oxidative and thermal stresses on stress granule formation in human induced pluripotent stem cells. PLoS One 12:e0182059. 10.1371/journal.pone.0182059 28746394 PMC5528897

[B119] Patel A. Lee H. O. Jawerth L. Maharana S. Jahnel M. Hein M. Y.. (2015). A liquid-to-solid phase transition of the ALS protein FUS accelerated by disease mutation. Cell 162, 1066–1077. 10.1016/j.cell.2015.07.047 26317470

[B120] Pilli M. Arko-Mensah J. Ponpuak M. Roberts E. Master S. Mandell M. A.. (2012). TBK-1 promotes autophagy-mediated antimicrobial defense by controlling autophagosome maturation. Immunity 37, 223–234. 10.1016/j.immuni.2012.04.015 22921120 PMC3428731

[B121] Piotrowska J. Hansen S. J. Park N. Jamka K. Sarnow P. Gustin K. E. (2010). Stable formation of compositionally unique stress granules in virus-infected cells. J. Virol. 84, 3654–3665. 10.1128/JVI.01320-09 20106928 PMC2838110

[B122] Pokrishevsky E. Grad L. I. Cashman N. R. (2016). TDP-43 or FUS-induced misfolded human wild-type SOD1 can propagate intercellularly in a prion-like fashion. Sci. Rep. 6:22155. 10.1038/srep22155 26926802 PMC4772009

[B123] Pokrishevsky E. Grad L. I. Yousefi M. Wang J. Mackenzie I. R. Cashman N. R. (2012). Aberrant localization of FUS and TDP43 is associated with misfolding of SOD1 in amyotrophic lateral sclerosis. PLoS One 7:e35050. 10.1371/journal.pone.0035050 22493728 PMC3320864

[B124] Prasad A. Bharathi V. Sivalingam V. Girdhar A. Patel B. K. (2019). Molecular mechanisms of TDP-43 misfolding and pathology in amyotrophic lateral sclerosis. Front. Mol. Neurosci. 12:25. 10.3389/fnmol.2019.00025 30837838 PMC6382748

[B125] Protter D. S. W. Parker R. (2016). Principles and Properties of Stress Granules. Trends Cell Biol. 26, 668–679. 10.1016/j.tcb.2016.05.004 27289443 PMC4993645

[B126] Ragagnin A. M. G. Shadfar S. Vidal M. Jamali M. S. Atkin J. D. (2019). Motor neuron susceptibility in ALS/FTD. Front. Neurosci. 13:532. 10.3389/fnins.2019.00532 31316328 PMC6610326

[B127] Rawal C.C. Zardoni L. Di Terlizzi M. Galati E. Brambati A. Lazzaro F.. (2020). Senataxin ortholog sen1 limits DNA:RNA hybrid accumulation at DNA double-strand breaks to control end resection and repair fidelity. Cell Rep. 31:107603. 10.1016/j.celrep.2020.107603 32375052

[B128] Reber S. Stettler J. Filosa G. Colombo M. Jutzi D. Lenzken S. C.. (2016). Minor intron splicing is regulated by FUS and affected by ALS-associated FUS mutants. EMBO J. 35, 1504–1521. 10.15252/embj.201593791 27252488 PMC4946139

[B129] Rhoads S. N. Monahan Z. T. Yee D. S. Leung A. Y. Newcombe C. G. O’Meally R. N.. (2018). The prionlike domain of FUS is multiphosphorylated following DNA damage without altering nuclear localization. Mol. Biol. Cell 29, 1786–1797. 10.1091/mbc.E17-12-0735 29897835 PMC6085830

[B130] Rojas P. Ramirez A. I. Fernandez-Albarral J. A. Lopez-Cuenca I. Salobrar-Garcia E. Cadena M.. (2020). Amyotrophic lateral sclerosis: a neurodegenerative motor neuron disease with ocular involvement. Front. Neurosci. 14:566858. 10.3389/fnins.2020.566858 33071739 PMC7544921

[B131] Rothblum-Oviatt C. Wright J. Lefton-Greif M. A. McGrath-Morrow S. A. Crawford T. O. Lederman H. M. (2016). Ataxia telangiectasia: a review. Orphanet J. Rare Dis. 11:159. 10.1186/s13023-016-0543-7 27884168 PMC5123280

[B132] Rulten S. L. Rotheray A. Green R. L. Grundy G. J. Moore D. A. Gomez-Herreros F.. (2014). PARP-1 dependent recruitment of the amyotrophic lateral sclerosis-associated protein FUS/TLS to sites of oxidative DNA damage. Nucleic Acids Res. 42, 307–314. 10.1093/nar/gkt835 24049082 PMC3874156

[B133] Ryu H. H. Jun M. H. Min K. J. Jang D. J. Lee Y. S. Kim H. K.. (2014). Autophagy regulates amyotrophic lateral sclerosis-linked fused in sarcoma-positive stress granules in neurons. Neurobiol. Aging 35, 2822–2831. 10.1016/j.neurobiolaging.2014.07.026 25216585

[B134] Salton M. Lerenthal Y. Wang S. Y. Chen D. J. Shiloh Y. (2010). Involvement of matrin 3 and SFPQ/NONO in the DNA damage response. Cell Cycle 9, 1568–1576. 10.4161/cc.9.8.11298 20421735

[B135] Sasagawa K. Matsudo Y. Kang M. Fujimura L. Iitsuka Y. Okada S.. (2002). Identification of Nd1, a novel murine kelch family protein, involved in stabilization of actin filaments. J. Biol. Chem. 277, 44140–44146. 10.1074/jbc.M202596200 12213805

[B136] Savitsky K. Bar-Shira A. Gilad S. Rotman G. Ziv Y. Vanagaite L.. (1995). A single ataxia telangiectasia gene with a product similar to PI-3 kinase. Science 268, 1749–1753. 10.1126/science.7792600 7792600

[B137] Sfeir A. Symington L. S. (2015). Microhomology-mediated end joining: a back-up survival mechanism or dedicated pathway. Trends Biochem. Sci. 40, 701–714. 10.1167/iovs.62.14.24 26439531 PMC4638128

[B138] Shen W. C. Li H. Y. Chen G. C. Chern Y. Tu P. H. (2015). Mutations in the ubiquitin-binding domain of OPTN/optineurin interfere with autophagy-mediated degradation of misfolded proteins by a dominant-negative mechanism. Autophagy 11, 685–700. 10.4161/auto.36098 25484089 PMC4502753

[B139] Shi K. Y. Mori E. Nizami Z. F. Lin Y. Kato M. Xiang S.. (2017). Toxic PRn poly-dipeptides encoded by the C9orf72 repeat expansion block nuclear import and export. Proc. Natl. Acad. Sci. U S A 114, E1111–E1117. 10.1073/pnas.1620293114 28069952 PMC5320981

[B140] Singatulina A. S. Hamon L. Sukhanova M. V. Desforges B. Joshi V. Bouhss A.. (2019). PARP-1 Activation Directs FUS to DNA damage sites to form PARG-reversible compartments enriched in damaged DNA. Cell Rep. 27, 1809–1821.e5. 10.1016/j.celrep.2019.04.031 31067465

[B141] Smeyers J. Banchi E. G. Latouche M. (2021). C9ORF72: what it is, what it does and why it matters. Front. Cell. Neurosci. 15:661447. 10.3389/fncel.2021.661447 34025358 PMC8131521

[B142] Spies J. Waizenegger A. Barton O. Surder M. Wright W. D. Heyer W. D.. (2016). Nek1 regulates Rad54 to orchestrate homologous recombination and replication fork stability. Mol. Cell 62, 903–917. 10.1016/j.molcel.2016.04.032 27264870 PMC5503685

[B143] Stoica R. De Vos K. J. Paillusson S. Mueller S. Sancho R. M. Lau K. F.. (2014). ER-mitochondria associations are regulated by the VAPB-PTPIP51 interaction and are disrupted by ALS/FTD-associated TDP-43. Nat Commun 5:3996. 10.1038/ncomms4996 24893131 PMC4046113

[B144] Stoica R. Paillusson S. Gomez-Suaga P. Mitchell J. C. Lau D. H. Gray E. H.. (2016). ALS/FTD-associated FUS activates GSK-3beta to disrupt the VAPB-PTPIP51 interaction and ER-mitochondria associations. EMBO Rep. 17, 1326–1342. 10.15252/embr.201541726 27418313 PMC5007559

[B145] Su Y. Ming G. L. Song H. (2015). DNA damage and repair regulate neuronal gene expression. Cell Res. 25, 993–994. 10.1038/cr.2015.85 26183066 PMC4559811

[B146] Sui J. Lin Y. F. Xu K. Lee K. J. Wang D. Chen B. P. (2015). DNA-PKcs phosphorylates hnRNP-A1 to facilitate the RPA-to-POT1 switch and telomere capping after replication. Nucleic. Acids Res. 43, 5971–5983. 10.1093/nar/gkv539 25999341 PMC4499152

[B147] Sun Y. Curle A. J. Haider A. M. Balmus G. (2020). The role of DNA damage response in amyotrophic lateral sclerosis. Essays Biochem. 64, 847–861. 10.1042/EBC20200002 33078197 PMC7588667

[B148] Ticozzi N. Vance C. Leclerc A. L. Keagle P. Glass J. D. McKenna-Yasek D.. (2011). Mutational analysis reveals the FUS homolog TAF15 as a candidate gene for familial amyotrophic lateral sclerosis. Am. J. Med. Genet. B Neuropsychiatr. Genet. 156B, 285–290. 10.1002/ajmg.b.31158 21438137

[B149] Tran M. Reddy P. H. (2020). Defective autophagy and mitophagy in aging and Alzheimer’s disease. Front. Neurosci. 14:612757. 10.3389/fnins.2020.612757 33488352 PMC7820371

[B150] Trenner A. Sartori A. A. (2019). Harnessing DNA double-strand break repair for cancer treatment. Front. Oncol. 9:1388. 10.3389/fonc.2019.01388 31921645 PMC6921965

[B151] Truong L. N. Li Y. Shi L. Z. Hwang P. Y. He J. Wang H.. (2013). Microhomology-mediated End Joining and Homologous Recombination share the initial end resection step to repair DNA double-strand breaks in mammalian cells. Proc. Natl. Acad. Sci. U S A 110, 7720–7725. 10.1073/pnas.1213431110 23610439 PMC3651503

[B152] Tsai Y. L. Coady T. H. Lu L. Zheng D. Alland I. Tian B.. (2020). ALS/FTD-associated protein FUS induces mitochondrial dysfunction by preferentially sequestering respiratory chain complex mRNAs. Genes Dev. 34, 785–805. 10.1101/gad.335836.119 32381627 PMC7263147

[B153] Tsang C. K. Liu Y. Thomas J. Zhang Y. Zheng X. F. (2014). Superoxide dismutase 1 acts as a nuclear transcription factor to regulate oxidative stress resistance. Nat. Commun. 5:3446. 10.1038/ncomms4446 24647101 PMC4678626

[B154] Vaidya A. Mao Z. Tian X. Spencer B. Seluanov A. Gorbunova V. (2014). Knock-in reporter mice demonstrate that DNA repair by non-homologous end joining declines with age. PLoS Genet. 10:e1004511. 10.1371/journal.pgen.1004511 25033455 PMC4102425

[B155] Van Mossevelde S. van der Zee J. Cruts M. Van Broeckhoven C. (2017). Relationship between C9orf72 repeat size and clinical phenotype. Curr. Opin. Genet. Dev. 44, 117–124. 10.1016/j.gde.2017.02.008 28319737

[B156] van Rheenen W. Shatunov A. Dekker A. M. McLaughlin R. L. Diekstra F. P. Pulit S. L.. (2016). Genome-wide association analyses identify new risk variants and the genetic architecture of amyotrophic lateral sclerosis. Nat. Genet. 48, 1043–1048. 10.1038/ng.3622 27455348 PMC5556360

[B157] Vance C. Al-Chalabi A. Ruddy D. Smith B. N. Hu X. Sreedharan J.. (2006). Familial amyotrophic lateral sclerosis with frontotemporal dementia is linked to a locus on chromosome 9p13.2–21.3. Brain 129, 868–876. 10.1093/brain/awl030 16495328

[B158] Vanden Broeck L. Callaerts P. Dermaut B. (2014). TDP-43-mediated neurodegeneration: towards a loss-of-function hypothesis? Trends Mol. Med. 20, 66–71. 10.1016/j.molmed.2013.11.003 24355761

[B159] Vijayakumar U. G. Milla V. Cynthia Stafford M. Y. Bjourson A. J. Duddy W. Duguez S. M. (2019). A systematic review of suggested molecular strata, biomarkers and their tissue sources in ALS. Front. Neurol. 10:400. 10.3389/fneur.2019.00400 31139131 PMC6527847

[B160] Walker C. El-Khamisy S. F. (2018). Perturbed autophagy and DNA repair converge to promote neurodegeneration in amyotrophic lateral sclerosis and dementia. Brain 141, 1247–1262. 10.1093/brain/awy076 29584802 PMC5917746

[B161] Walker C. Herranz-Martin S. Karyka E. Liao C. Lewis K. Elsayed W.. (2017). C9orf72 expansion disrupts ATM-mediated chromosomal break repair. Nat. Neurosci. 20, 1225–1235. 10.1038/nn.4604 28714954 PMC5578434

[B162] Wang H. Adhikari S. Butler B. E. Pandita T. K. Mitra S. Hegde M. L. (2014). A perspective on chromosomal double strand break markers in mammalian cells. Jacobs J. Radiat. Oncol. 1:003. 25614903 PMC4299656

[B169] Wang P. Deng J. Dong J. Liu J. Bigio E. H. Mesulam M.. (2019). TDP-43 induces mitochondrial damage and activates the mitochondrial unfolded protein response. PLoS Genet. 15:e1007947. 10.1371/journal.pgen.1007947 31100073 PMC6524796

[B163] Wang H. Dharmalingam P. Vasquez V. Mitra J. Boldogh I. Rao K. S.. (2017). Chronic oxidative damage together with genome repair deficiency in the neurons is a double whammy for neurodegeneration: is damage response signaling a potential therapeutic target. Mech. Ageing Dev. 161, 163–176. 10.1016/j.mad.2016.09.005 27663141 PMC5316312

[B168] Wang I. F. Guo B. S. Liu Y. C. Wu C. C. Yang C. H. Tsai K. J.. (2012). Autophagy activators rescue and alleviate pathogenesis of a mouse model with proteinopathies of the TAR DNA-binding protein 43. Proc. Natl. Acad. Sci. U S A 109, 15024–15029. 10.1073/pnas.1206362109 22932872 PMC3443184

[B164] Wang H. Guo W. Mitra J. Hegde P. M. Vandoorne T. Eckelmann B. J.. (2018). Mutant FUS causes DNA ligation defects to inhibit oxidative damage repair in amyotrophic lateral sclerosis. Nat. Commun. 9:3683. 10.1038/s41467-018-06111-6 30206235 PMC6134028

[B165] Wang H. Hegde M. L. (2019). New mechanisms of DNA repair defects in fused in sarcoma-associated neurodegeneration: stage set for DNA repair-based therapeutics. J. Exp. Neurosci. 13:1179069519856358. 10.1177/1179069519856358 31217692 PMC6558540

[B170] Wang W. Y. Pan L. Su S. C. Quinn E. J. Sasaki M. Jimenez J. C.. (2013). Interaction of FUS and HDAC1 regulates DNA damage response and repair in neurons. Nat. Neurosci. 16, 1383–1391. 10.1038/nn.3514 24036913 PMC5564396

[B166] Wang H. Rangaswamy S. Kodavati M. Mitra J. Guo W. Guerrero E. N.. (2019). RT(2) PCR array screening reveals distinct perturbations in DNA damage response signaling in FUS-associated motor neuron disease. Mol. Brain 12:103. 10.1186/s13041-019-0526-4 31801573 PMC6894127

[B167] Wang H. Zhao A. Chen L. Zhong X. Liao J. Gao M.. (2009). Human RIF1 encodes an anti-apoptotic factor required for DNA repair. Carcinogenesis 30, 1314–1319. 10.1093/carcin/bgp136 19483192 PMC2718077

[B171] Watanabe Y. Nakagawa T. Akiyama T. Nakagawa M. Suzuki N. Warita H.. (2020). An amyotrophic lateral sclerosis-associated mutant of C21ORF2 is stabilized by NEK1-mediated hyperphosphorylation and the inability to bind FBXO3. iScience 23:101491. 10.1016/j.isci.2020.101491 32891887 PMC7481237

[B172] Wheeler J. R. Matheny T. Jain S. Abrisch R. Parker R. (2016). Distinct stages in stress granule assembly and disassembly. eLife 5:e18413. 10.7554/eLife.18413 27602576 PMC5014549

[B173] Wolozin B. Ivanov P. (2019). Stress granules and neurodegeneration. Nat. Rev. Neurosci. 20, 649–666. 10.1038/s41583-019-0222-5 31582840 PMC6986315

[B174] Wu C. C. Jin L. W. Wang I. F. Wei W. Y. Ho P. C. Liu Y. C.. (2020). HDAC1 dysregulation induces aberrant cell cycle and DNA damage in progress of TDP-43 proteinopathies. EMBO Mol. Med. 12:e10622. 10.15252/emmm.201910622 32449313 PMC7278561

[B175] Yu C. H. Davidson S. Harapas C. R. Hilton J. B. Mlodzianoski M. J. Laohamonthonkul P.. (2020). TDP-43 triggers mitochondrial DNA release *via* mPTP to activate cGAS/STING in ALS. Cell 183, 636–649.e18. 10.1016/j.cell.2020.09.020 33031745 PMC7599077

[B176] Zago S. Poletti B. Morelli C. Doretti A. Silani V. (2011). Amyotrophic lateral sclerosis and frontotemporal dementia (ALS-FTD). Arch. Ital. Biol. 149, 39–56. 10.4449/aib.v149i1.1263 21412715

[B177] Zhang X. Wang F. Hu Y. Chen R. Meng D. Guo L.. (2020). *in vivo* stress granule misprocessing evidenced in a FUS knock-in ALS mouse model. Brain 143, 1350–1367. 10.1093/brain/awaa076 32358598

[B178] Zhou B. Huang C. Yang J. Lu J. Dong Q. Sun L. Z. (2009). Preparation of heteroduplex enhanced green fluorescent protein plasmid for *in vivo* mismatch repair activity assay. Anal. Biochem. 388, 167–169. 10.1016/j.ab.2009.02.020 19248754 PMC2670966

